# Expression of hypoxia-inducible genes is suppressed in altered gravity due to impaired nuclear HIF1α accumulation

**DOI:** 10.1038/s41598-023-41686-1

**Published:** 2023-09-04

**Authors:** Mostafa A. Aboouf, Cora S. Thiel, Sergey M. Borisov, Svantje Tauber, Eva Bönzli, Nelli Schetle, Oliver Ullrich, Max Gassmann, Johannes Vogel

**Affiliations:** 1 Vetsuisse Faculty, Institute of Veterinary Physiology, University of Zurich, Winterthurerstrasse 260, 8057 Zurich, Switzerland; 2 Zurich Center for Integrative Human Physiology (ZIHP), University of Zurich, Zurich, Switzerland; 3 Vetsuisse Faculty, Center for Clinical Studies, University of Zurich, 8057 Zurich, Switzerland; 4 Department of Biochemistry, Faculty of Pharmacy, Ain Shams University, Cairo, 11566 Egypt; 5 Faculty of Medicine, Institute of Anatomy, University of Zurich, Winterthurerstrasse 190, 8057 Zurich, Switzerland; 6 UZH Space Hub, Air Force Center, Air Base Dübendorf, Überlandstrasse 270, 8600 Dubendorf, Switzerland; 7 Institute of Analytical Chemistry and Food Chemistry, Graz University of Technology, Stremayrgasse 9, 8010 Graz, Austria; 8 Clinical Laboratory, Department for Clinical Services and Diagnostics, Vetsuisse Faculty, University of Zurich, Winterthurerstrasse 260, 8057 Zurich, Switzerland

**Keywords:** Cell biology, Physiology, Transcriptional regulatory elements

## Abstract

Extravehicular activities, the backbone of manned space exploration programs, set astronauts into mild hypoxia. Unfortunately, microgravity aggravates threatening symptoms of hypoxia such as vision impairment and brain edema. Hypoxia-inducible factors (HIFs) sense cellular hypoxia and, subsequently, change the cells’ expression profile instantaneously by rapidly translocating—most likely cytoskeleton-dependently—into the nucleus and subsequently forming transcription complexes with other proteins. We tested the hypothesis that this fundamental process could be altered by sudden changes in gravitational forces in parabolic flights using a newly developed pocket-size cell culture lab that deoxygenizes cells within 15 min. Sudden gravity changes (SGCs 1g–1.8g–0g–1.8g–1g) during hypoxic exposure suppressed expression of the HIF1α-dependent genes investigated as compared with hypoxia at constant 1g. Normoxic cells subjected to SGCs showed reduced nuclear but not cytoplasmatic HIF1α signal and appeared to have disturbed cytoskeleton architecture. Inhibition of the actin-dependent intracellular transport using a combination of myosin V and VI inhibitors during hypoxia mimicked the suppression of the HIF1α-dependent genes observed during hypoxic exposure during SGCs. Thus, SGCs seem to disrupt the cellular response to hypoxia by impairing the actin-dependent translocation of HIF1α into the nucleus.

## Introduction

Extravehicular activities (EVAs) are indispensable for manned space exploration programs. However, an old and well-known fundamental challenge is the reduced mobility of the astronauts due to the pressurization of space suits necessary for such missions. Thus, it has been recommended to reduce the pressure inside the space suits to about 60% of sea-level atmospheric pressure while simultaneously increasing the atmospheric oxygen concentration to about 32% (https://ntrs.nasa.gov/citations/20150021491). Alternatively, raising the oxygen concentration inside a space suit to 100% while simultaneously further reducing the suit pressure (although proposed) is quite problematic because of the heightened flammability and oxygen toxicity risks. Altogether, today’s favored recommendation is a compromise that exposes the astronaut to mildly reduced oxygen concentrations (mild hypoxia of 10–15% reduced oxygen partial pressure) at a suit pressure low enough to allow an acceptable degree of mobility. Unfortunately, microgravity and reduced atmospheric pressure (hypobaria) aggravate threatening symptoms of hypoxia^1^, such as vision impairment, edema of the visual nerve papilla, and increased intracranial pressure. This symptoms complex is called VIIP-Syndrome (Visual Impairment/Intracranial Pressure syndrome) and is the major human system risk in the International Space Station program. Moreover, these symptoms are often associated with headache, nausea, vomiting, sleep disturbance, and poor physical performance. The latter combination of symptoms is also typical for acute mountain sickness (AMS) that occurs after rapid ascent to high altitudes^2^. Although the detailed pathophysiology of VIIP and AMS is still not fully understood, a large body of evidence suggests a dysregulated cellular response to hypoxia is involved^3^.

Altitude-induced hypoxia is a regular physiological challenge in military aviation with high-performance combat aircraft, in which the limits of the human body's physical and mental performance are quickly reached^4,5^. Hypoxia leads to many performance and flight safety impairing symptoms and consequences, highlighting the importance of specific problem detection, training, and countermeasures^6^. The interplay of rapid gravity changes and the cellular consequences of hypoxia is particularly relevant because of the maneuvers flown in military aviation but has been studied only rudimentarily at the cellular level. For example, we recently identified that transcriptional effects in different gravity conditions occur extremely rapidly on a second- to minute-scale in different cell types^7,8^ and affected hypoxia-inducible factor (HIF-1α)-dependent transcripts^9^. The switch from micro- to hypergravity conditions also potentiated antigen-induced immune cell activation and cytokine secretion^10^. This “priming,” i.e., increased sensitivity to stimuli, was demonstrated in a series of parabolic flight experiments with polymorphonuclear leukocytes^11,12^. Therefore, studying the combination of gravity changes and cellular hypoxia consequences is an important topic for aerospace medicine.

Since higher animal life, especially that of mammals including man, is crucially dependent on oxygen to fuel their highly active metabolism, a shortage of oxygen is an alarming situation for any mammalian cell. Consequently, evolution developed a rapid and highly precise cellular oxygen sensor^13^ capable of changing the expression profile in hypoxic cells instantaneously^14^ based on proteins, called HIFs, which are continuously produced and degraded in the presence of oxygen. During hypoxia, either HIF1α or HIF2α, the two master regulators of the cellular response to oxygen shortage, are stabilized in the cytoplasm and translocated to the nucleus to form transcription complexes with other proteins and finally bind to the hypoxia response element (HRE) located in the promoter regions of oxygen-dependent genes. In the last decades, a huge number of oxygen-dependent genes have been identified, underlining the biological importance of this fundamental process. Of note, regulation of oxygen-dependent genes occurs almost continuously somewhere in our body and is important in key physiological processes such as angiogenesis (formation of new capillaries), regulation of red blood cell mass, wound healing, or fighting infections^15,16^. Regarding the symptoms of VIIP and AMS, the oxygen-regulated gene vascular endothelial growth factor (VEGF) is of central interest as being up-regulated by rather mild tissue hypoxia^17^. Originally after its discovery, VEGF was termed “vascular permeability factor” as the first step of its action to stimulate the angiogenesis in hypoxic tissues is to weaken the mechanical interactions between endothelial cells^18,19^. This has unfavorable consequences especially in the brain and other neuronal tissues such as the retina and larger nerves such as the optic nerve because the disruption of cell–cell interaction of brain endothelial cells weakens the blood–brain barrier. Consequently, extravasation of blood plasma and water occurs leading to the clinical symptoms of brain edema and increased intracranial pressure with subsequent impairment of brain function such as nausea or vomiting, vision impairment, and headache as typically seen in VIIP and AMS. Regarding the health of astronauts, it is worth mentioning that also the function of the immune system is reduced by hypoxia and that this might also be aggravated by other environmental factors inherent to space flights^20–22^.

As the response to hypoxia requires a rapid nuclear transfer of the stabilized HIFs, which appears to be dependent on the cytoskeleton^23^ and complex formation with other proteins of different densities to trigger the subsequent regulation of the cellular “emergency” response to hypoxia, it is tempting to speculate that this process could be altered by sudden changes of gravitational forces. This hypothesis is in line with the fact that vesicles, RNA, and proteins are actively transported within cells along the cytoskeleton. There are two transport systems of which the first is based on actin filaments with the myosin protein family as the motors. When myosin IV is attached to the cargo, it will be transported to the pointed end, while when myosin V is attached to the cargo, it will be transported to the barbed end. The second transport system uses microtubules with dynein and kinesin as the motors transporting the cargo either to the minus or the plus end, respectively. The transport speed and probability for detachment from the track are functions of the weight of the cargo and the number of motors. In general, two or three, maximally five motors carry one cargo^24^.

Because hypoxia modulates many crucial biological processes, insights into the cellular response to hypoxia during altered gravity are of utmost significance for future manned space flights, such as interplanetary flights or missions on the moon, that take months to years and include excessive EVAs at hypobaric hypoxia in combination with microgravity.

## Materials and methods

### Cell culture

We used MDA-MB-468 human epithelial adenocarcinoma-derived breast cancer cells (ATCC HTB-132™, Manassas, VA, USA) because they are known to reduce proliferation and survive severe hypoxia^25^ and to be consistent with our previous work^9^. The MDA-MB-468 cells were cultured in Dulbecco’s Modified Eagle’s Medium (DMEM) containing high glucose levels (4.5 g/l) (Gibco/Life Technologies, Hessen, Germany) and supplemented with 10% Fetal Bovine Serum (10270-106, Gibco/Life Technologies, Hessen, Germany) and 1% penicillin/streptomycin (Gibco/Life Technologies, Hessen, Germany). After initial expansion for 48 h, the cells were seeded for the normoxic experiments (37 °C, room air with 5% CO_2_).

In further experiments, inhibition of intracellular motor proteins was performed in hypoxic (0.2% O_2_) versus normoxic (21% O_2_) cultures of MDA-MB-468 cells. To this end, cells were cultured for 48 h to 70% confluency and then treated with either 50 µM Myovin1 (specific Myosin V inhibitor, Merck Millipore, 475984), 10 µM 2,4,6-triiodophenol (specific Myosin VI inhibitor, Sigma Aldrich, 137723) or a combination of both inhibitors. Moreover, 20 µM Ciliobrevin D (specific Dynein inhibitor, Merck Millipore, 250401) or 1 µM dimethylenastron (specific Kinesin inhibitor, Merck Millipore, 324622), or a combination of both inhibitors were tested on the cells. All treatments were performed for 60 min versus a vehicle control before either harvesting the cells for RNA analysis or fixing for immunocytochemistry as described below.

### Pocket-size cell culture devices

**Figure 1:**
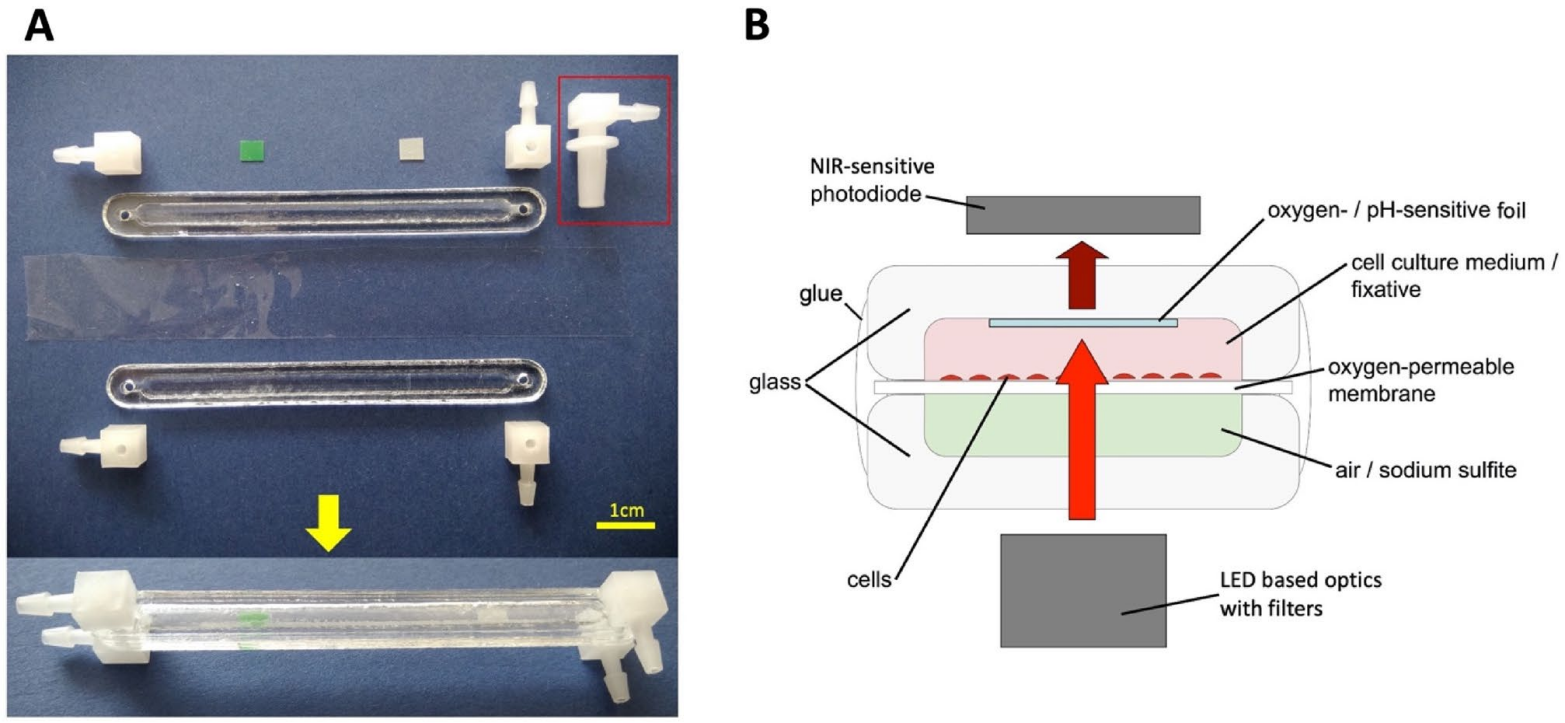
Specifically designed oxygen-tight cell culture assemblies for the hypoxia cell culture devices. Panel (**A**) shows the individual parts (above the arrow) and the final assembled appearance (below the arrow). Panel (**B**) shows a schematic cross-section through the assembled cell culture chamber. It consists of two glass stripes that have been etched using hydrofluoric acid and glued together with an oxygen-permeable membrane in between, in a way to form two separate compartments (pink and light green in **B**) separated by that membrane. Each of the compartments has a height of about 1 mm to minimize shear stress on the cells when changing fluids. In addition, each compartment has an inlet and outlet made of Elbow Luer-lock connectors (red box in **A**) to allow connecting syringes with silicone tubes, Y-tube fittings, and female Luer-lock connectors (https://ibidi.com/search?controller=search&page=2&s=flow+accessories). The upper compartment contains the oxygen- (green square in **A**) and pH-sensitive (white square in **A**) sensor foils. After assembling, the cell culture chambers have been sterilized carefully with 3% hydrogen peroxide and coated with Poly-L-Lysine (P4707 Sigma-Aldrich) before seeding the cells into the upper compartment (pink in **B**). The lower compartment initially contains air and is later filled with sodium sulfite-based deoxygenation solution (see supplemental material). The cell culture assemblies fit between the LED-based optics (light is indicated by red [excitation] and dark red [emission] arrows) and the photodiode on the LIA board of the cell culture device (version 2) for hypoxic experiments. For details on the optics refer to the supplemental material.

We designed, constructed, and realized two different types of pocket-size cell culture devices, one, version 1, for normoxic experiments, and the other, version 2, for hypoxic experiments (for details see supplemental material Figs. S1–S12). Briefly, in the normoxic experiments, cells were seeded on IBIDI slides (µ-Slide I Luer, 0.8 mm chamber height, IBIDI) previously coated with poly-L-Lysine (P4707, Sigma-Aldrich). Since oxygen diffuses well through polystyrene, the material of the IBIDI slides (not shown), and consequently hypoxic conditions cannot be reached with IBIDI slides it was necessary to design and manufacture oxygen-tight cell culture chambers for the hypoxic experiments (Fig. 1). For that purpose, these cell culture assemblies consist of two compartments that are made of glass and separated by a water-tight, oxygen-permeable plastic membrane (20 µm thick, Lumox-foil, Sarstedt, product code: 94.6077.317). This generates an upper and a lower compartment that both have an inlet as well as an outlet to exchange fluids. The upper compartment contains two pieces of luminescent foil one enables optical sensing of pO_2_^26^ the other of pH^27^ (for details see supplemental material). The hypoxic cell culture assemblies (Fig. 1) allow setting cells to near anoxia within about 15 min. For the final experiments, the cells were cultured to a confluency of 60–70%, and the medium exchange was performed every 48 h. Cells were either fixed for staining or harvested for further molecular analysis as described below.

Each of the cell culture devices (Figs. S3–S8) can hold six separate cell cultures either inside IBIDI slides (normoxic experiments) or inside oxygen-tight glass cell culture assemblies (hypoxic experiments). Both types of cell culture devices control and record temperature as well as record linear and angular acceleration forces in all three space axes on a micro-SD card. The more complex type of these cell culture devices allows additional recording of clock time, atmospheric pressure, O_2_ concentration, and pH as an overall measure for the condition of the cell cultures in the six separate cell culture experiments. All data can also be sent via Bluetooth to an Android device. In addition, the cell culture devices allow fluid exchange at any desired time point simultaneously in all six cell cultures with the aid of two separate levers. The simpler cell culture device was used for normoxic experiments. For immunocytochemistry, the six IBIDI slides contained in each device were equipped with two 1 mL syringes containing a fixing solution (4% formalin) (see supplemental material). The more complex cell culture device containing six of the oxygen-tight cell culture assemblies was used for the hypoxic experiments. Again, each of the oxygen-tight cell culture assemblies was connected to two 1 mL syringes. This allows fluid exchange separately in the upper (cells with medium –> fixing solution for later analysis) as well as the lower compartment (air –> deoxygenation solution). For more details on the composition of these solutions, see the supplemental material.

We participated in the 4th Swiss parabolic flight campaign (SPFC) with six of the simpler devices for normoxic experiments and six of the more complex cell culture devices for hypoxic experiments.

### Altered gravity

**Figure 2:**
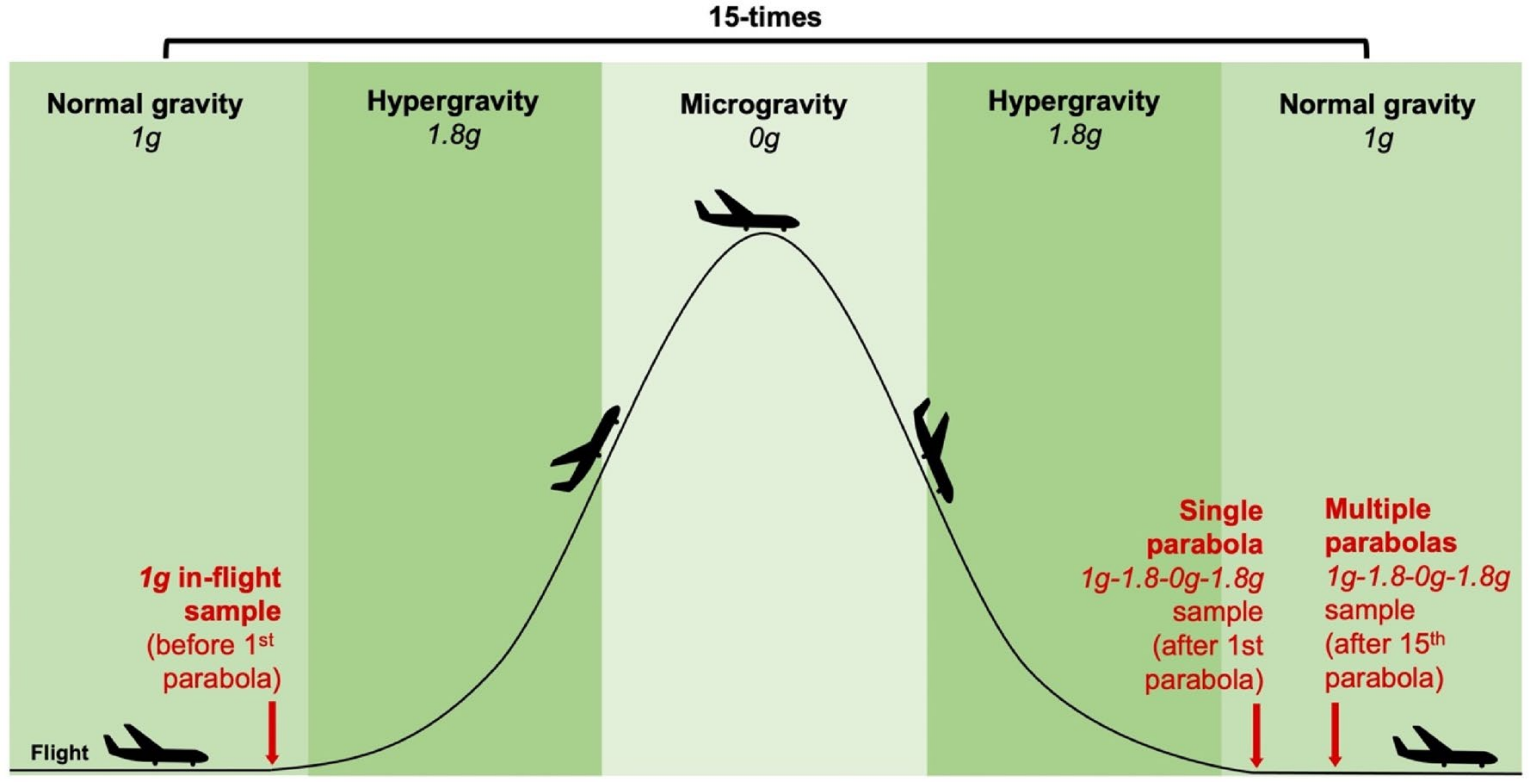
Scheme of the parabolic flight maneuver and schedule of sampling the biological material (red text and arrows). From normal flight with 1g, the aircraft is accelerating and then rises steeply into the sky, which induces 1.8g gravity for about 20 s. Thereafter, the aircraft’s engines are almost stopped which induces microgravity, in this case, 0g for about 22 s. To follow the shape of the parabola, the aircraft must perform elevator movements as indicated. To end the 0g phase, the aircraft is pulled out which again induces hypergravity of 1.8g till the aircraft is flying again normally, which re-establishes 1g. This cycle is repeated 15 times.

Parabolic flights offer a repetitive sequence of SGCs from 1g to 1.8g to 0g and back to 1.8g and 1g at a precision of 10^−2^g to 10^−3^g on board the Airbus A310 ZERO-G (reg. no. F-WNOV) operated by Novespace (https://www.airzerog.com/). Data shown here were obtained in the 2nd and mainly in the 4th SPFC (https://www.youtube.com/watch?v=sbkWR2EiR-I). Each campaign consists of 15 parabolas of 0g (as explained above) followed by one or two parabolas each of 0.38g (Mars gravity) as well as 0.16g (Moon gravity). However, all biological experiments were terminated after 15 parabolas of 0g (Fig. 2) whereas the recording of physical data was stopped always after the last parabola. All biological data presented here are from the 4th SPFC and 1g ground control samples as indicated.

### Parabolic flight experiments

**Figure 3:**
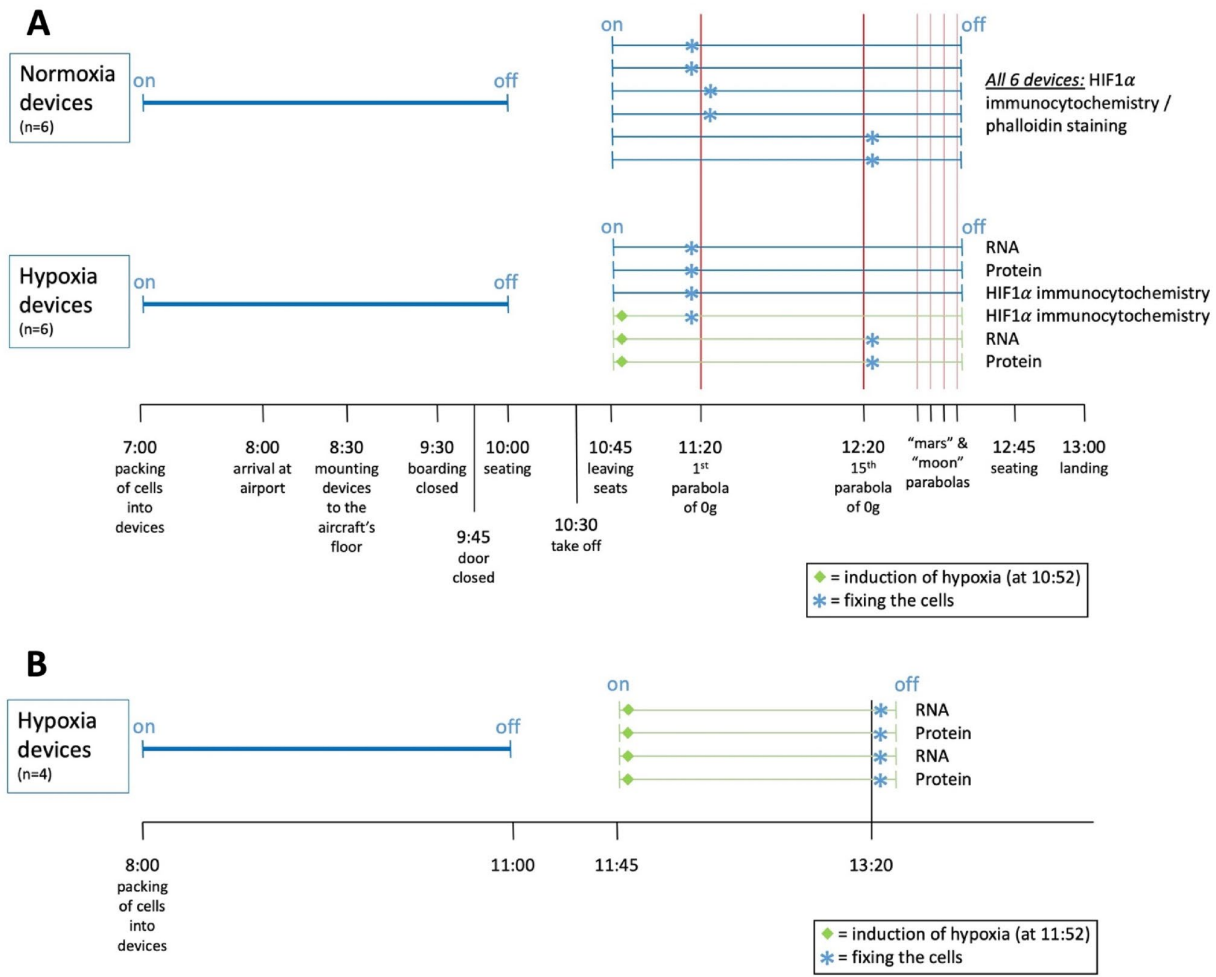
A detailed schedule for in-flight (**A**) and ground control (**B**) experiments. Cells were packed early in the morning into the cell culture devices, which were switched on and transported in a heat-insulated box to Dübendorf airport on the day of the parabolic flight (**A**) or placed on the lab bench for the ground control experiments (**B**). Three hours later the devices were switched off for 45 min (cf. text) at timepoint “seating”. Then all devices were switched on again at the time point “leaving seats”. Seven minutes later, hypoxia was induced in three hypoxia devices (green diamonds on green lines in **A**). For the ground control experiments (**B**) only hypoxia devices were used. Using the same time schedule as for the in-flight experiments, the cells were left inside the devices for 3 h, set into hypoxia seven minutes after switching the devices on again after a switch-off period of 45 min, and finally fixed after another 95 min. The termination of the experiments is indicated by a blue star. The downstream analysis applied to the cells is indicated on the right side (RNA = RT-PCR, Protein = Western blotting against HIF1α).

After packing the cell culture assemblies into the devices in our laboratory, the heating elements in the devices were switched on to keep the cells at 37 °C. The devices were then transported in a heat-insulated box to the Air Base Dübendorf and mounted to the aircraft’s floor in a way that the SGCs were acting perpendicular to the cell’s host-surface that was orientated between cells and the Earth’s surface (see supplemental material). For safety reasons, we were not allowed to have our devices switched on from the moment of seating till reaching the final flight level and having the approval to leave the seats. Thus, the devices were switched off directly before seating and then switched on again immediately after leaving the seats. Consequently, the heating was off for approximately 45 min after the take-off resulting in a temperature drop in the cell culture devices (see supplemental material). Cell cultures inside three of the more complex containers were set to hypoxia about seven minutes after switching on the devices. It takes about 5 min to warm up these containers again to 37 °C (see supplemental material). The other three of the more complex devices were left normoxic. Then it took another 35 min to reach the area where the parabolas were flown (over the Mediterranean Sea). The experimental scheme is illustrated in Fig. 3A. Briefly, the termination of the six experiments inside the normoxia devices was as follows: two were terminated just before the 1st parabola, two after the 1st parabola, and two after the 15th parabola. The experiments inside three hypoxia devices with normoxic cell cultures as well as one with hypoxic cell cultures were terminated before the 1st parabola. The experiments inside the two remaining hypoxia devices with the hypoxic cell cultures were terminated after the 15th parabola. The experiments terminated before the 1st parabola served as in-flight controls, those terminated after the 1st or after the 15th were used for further molecular analysis.

### Ground control experiments

After expansion in standard polystyrene culture dishes (12 cm diameter), the cells were trypsinized and seeded into our specifically designed, oxygen-tight cell culture assemblies (Fig. 1) and cultured to a confluency of 60–70%. The medium exchange was performed every 48 h. Two containers were defined to be used for RNA analysis and another two for protein analysis. To mimic the time schedule of the parabolic flights, the cells were mounted early in the morning into the hypoxia devices. Then the devices were switched on for 3 h and thereafter switched off for 45 min as in the aircraft (Fig. 3B). Seven minutes after switching the devices on again, hypoxia was induced by operating the respective lever of all containers that filled the lower compartment of all cell culture assemblies with the de-oxygenation solution. Ninety minutes later, the cells were fixed by operating the other lever in all cell culture devices for later RNA and protein analysis.

### Molecular analysis

The cell lysates were used either for Western blot against HIF1α or to perform RT-PCR for vascular endothelial growth factor A (VEGFA), Lactate Dehydrogenase A (LDHA), Egl-9 Family Hypoxia Inducible Factor 1 (EGLN1 or PHD2), glucose transporter type 1 (GLUT1), erythropoietin (EPO), CBP/p300 interacting transactivator with ED-rich tail 2 (CITED2), POU class 5 homeobox transcription factor (OCT4), and lastly β-actin (ACTB) as house-keeping gene. Both methods' details are described in the supplemental material.

Cells fixed with formalin were used for immunocytochemistry against HIF1α and phalloidin staining as described in the supplemental material. For quantification of the HIF1α staining intensities, image stacks for CY3-, ALEXA 488-, and DAPI-labeled cells were acquired using a fluorescence microscope (Axio-Imager, Zeiss). The illumination of the cells by the LED-based light source (aperture, gray filter) was carefully kept the same for all images. Then, by defining a graphics tool with a diameter of 2 µm diameter, the optical density of the cytoplasm directly nearby the nucleus as well as the optical density in the center of the nucleus, while avoiding the nucleolus, was determined for individual cells. In total, about 640 cells of 9–12 IBIDI slides per condition (in-flight 1g, after the 1st and after the 15th parabola) and about 170 cells for the cytoskeleton transport inhibition experiments were analyzed.

### Statistics

Data were analyzed using GraphPad Prism software (V9.5.0). Groups were compared using a one-way ANOVA followed by Bonferroni post hoc test. Data are presented as mean ± SD. Differences were considered statistically significant at *p* < 0.05.

## Results

### The devices’ performance check confirms the precision of the experimental parameters

Because we were not allowed to have our devices switched on while remaining seated during the take-off of the aircraft, the cells could not be kept at 37 °C for the about 45 min this took. However, when switching on the devices after leaving the seats, the temperature inside the devices used for the hypoxic experiments was still around 25 °C and 20 °C in those used for the normoxic experiments that were flown and handled simultaneously. Consequently, the normoxia devices reached a temperature of 37 °C in approximately 20 min, whereas the hypoxia devices were back at this temperature already after approximately 5 min (supplemental Fig. S9).

For measurement of the acceleration forces in all directions of space as well as temperature, both normoxic and hypoxic cell culture devices were equipped with the MPU-6050 (InvenSense). This chip is a MEMS (micro-electro-mechanical system) sensor that detects linear as well as angular acceleration forces with high accuracy so that next to the gravitational forces, linear acceleration forces in the nose-tail-direction at the beginning of each parabola were clearly detectable. Moreover, the elevator movements of the aircraft necessary to follow the parabolas were also clearly detectable (supplemental Figs. S10A, S10B).

In the devices used for the normoxic experiments, barometric pressure was measured using the MPL115A1 (NXP Semiconductors) that has an accuracy of ± 10 hPa and consequently could detect only the pressure difference in cabin pressure between ground and final flight level (101.56 ± 0.044 kPa vs. 91.74 ± 2.94 kPa). In contrast, the devices used for the hypoxic experiments were equipped with the LPS22HBTR (STMicroelectronics) with an accuracy of ± 0.1 hPa, thus, it is 100 times more sensitive. Therefore, we could detect the barometric pressure changes due to the deformation of the cabin because of changes in gravitational forces during the parabolic flight maneuver (supplemental Fig. S10C).

The response of the sensor foils obtained with compact home-made optoelectronics was compared to the calibrations generated for the same materials using a commercially available phase fluorometer from PyroScience (pO_2_ sensor, supplemental Fig. S11A) and Stanford Research lock-in amplifier equipped with a photomultiplier detector (pH sensor, Fig. S11B). The pO_2_ sensor showed very similar behavior with the two set-ups and the highest sensitivity from 0 to − 2 kPa pO_2_ which is advantageous for measurements at hypoxic conditions. In contrast, the pH sensor material that is a dual-lifetime referenced pH sensor^27^ did not deliver the same phase resolution as in the case of the commercial equipment. This was expected because we did not measure the decay of the reference dye since at this stage we wanted to keep the set-up simple, and we were more interested in the oxygen measurements. However, in preliminary experiments without using the reference signal (not shown), we found the best phase resolution for the pH measurements with a blue LED although the pH sensor was initially designed for excitation with red light (cf. supplemental material).

### The cellular response to hypoxia is impaired by altered gravity

**Figure 4:**
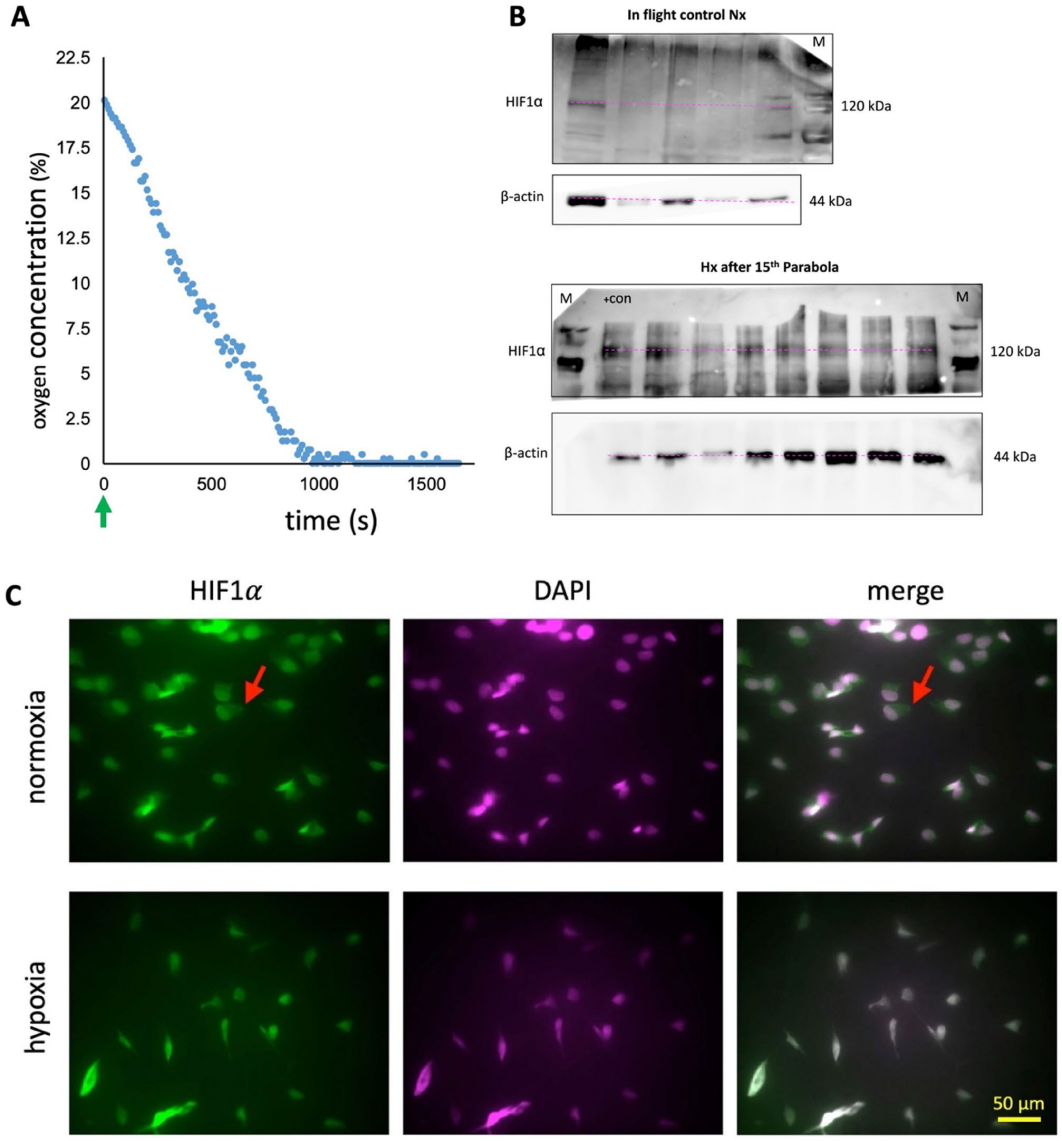
In-flight deoxygenation kinetic and quality. After switching on, the removal of oxygen in the cell cultures was initiated (green arrow) in the respective devices. Panel (**A**) shows the resulting oxygen profile. Near anoxia is reached after about 900 s (15 min) and stayed that low for the remaining time of recording or at least another 90 min (not shown). Note that the experiments were terminated latest 88 min after induction of hypoxia. (**B**) After the 15th parabola, protein, and RNA were extracted from the cells. It was not easy, but possible, to obtain enough protein from each glass cell culture chamber for Western blotting but it was sufficient to confirm HIF1α induction. Therefore, due to the limited sample volume, we had to cut the membrane before hybridization with primary antibodies at the corresponding band size, according to our initial testing and the antibody datasheet while avoiding stripping/re-staining the membranes. (M) is molecular weight marker ladder and (+ con) is positive control (**C**) After rinsing the lower compartment of the oxygen-tight cell culture chambers with sodium sulfite, the typical hypoxia-induced HIF1α accumulation in the cell nucleus (counterstained with DAPI) occurred, while staining in the cytoplasm (red arrows) decreased at the same time (magnification 40x, larger magnifications were not possible with our microscope because the cell culture chambers require a minimum distance between cells and objective lens of at least 2 mm).

During the parabolic flight and after flushing the lower compartment of the oxygen-tight cell culture assemblies, the oxygen in the cell cultures dropped rapidly to reach its lowest value around 15 min later. From that time on, the equipment recorded an oxygen concentration of 0.042 ± 0.1% till switching off the devices (Fig. 4A). Obtaining protein extract from glass cell culture assemblies, whose cell growth area is only 2.3 cm^2^, was challenging and not easy. Therefore, we were not able to extract enough protein for Western blotting from all cell culture assemblies used for this purpose. Nevertheless, the Western blot of representative samples (Fig. 4B) confirmed HIF1α induction by hypoxia. Additionally, immunocytochemistry staining showed the typical nuclear accumulation of HIF1α relative to the cytoplasm in hypoxic compared to normoxic cell cultures (Fig. 4C). These qualitative observations confirm the effective de-oxygenation and the precise measurements of oxygen concentration by the device.

**Figure 5:**
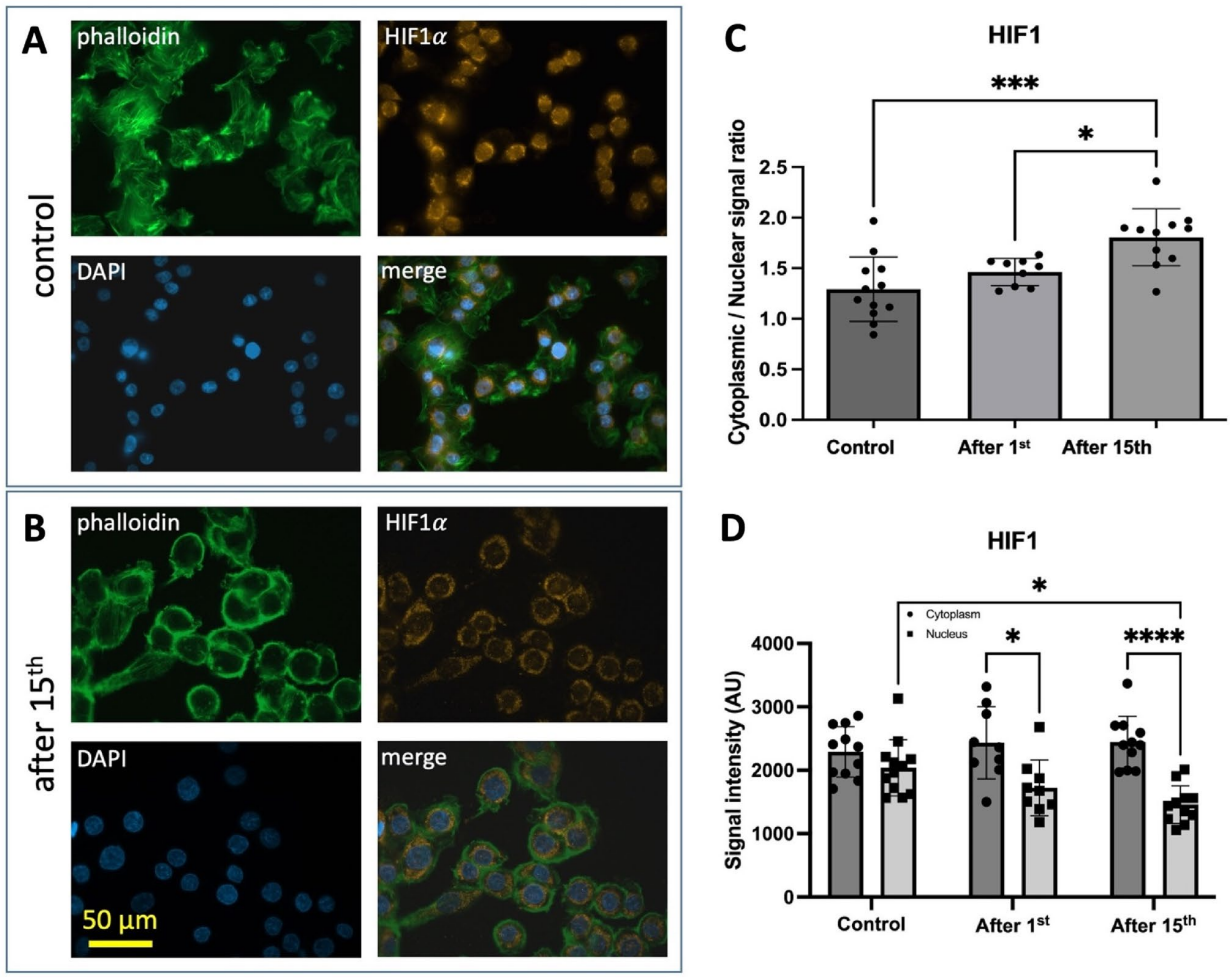
Effect of Sudden Gravity Changes on the subcellular HIF1α distribution in normoxic cells. Panels (**A**) and (**B**) show cells from normoxic containers (version 1) before any parabola (**A**) and after the 15th parabola (**B**) stained for actin cytoskeleton (phalloidin), HIF1α and nuclei (DAPI). Repeated parabolic flight maneuvers appear to reduce the staining intensity of the cytoskeleton and alter its structure integrity (phalloidin staining). Moreover, the subcellular HIF1α distribution appeared to be altered by the parabolic flight maneuvers. Quantification of the HIF1α staining intensity (**C** and **D**) revealed that after the 15th parabola, the cytoplasmic to nuclear ratio of the HIF1α staining intensity was significantly increased compared to the control (**C**). This was due to significantly reduced HIF1α nuclear staining intensity. The intensity of the cytoplasmic staining remained constant (**D**). Data are shown as Means ± SD of 9–12 independent experiments per condition * = *P* < 0.05, *** = *P* < 0.001, **** = *P* < 0.0001.

Next, we analyzed the cells from the normoxic containers (version 1) by performing HIF1α as well as cytoskeleton (phalloidin) staining. Nuclei were counterstained with DAPI (Fig. 5A). The staining intensity of the cytoskeleton appeared to be reduced and less structured after the 15th parabola (Fig. 5A vs. B). Moreover, the subcellular HIF1α distribution appeared to be altered by the parabolic flight maneuvers. Quantification of this observation showed that after the 15th parabola, the cytoplasmic to the nuclear ratio of the HIF1α staining intensity was significantly increased compared to the control or to that measured after the 1st parabola (Fig. 5C). This was due to significantly reduced nuclear HIF1α staining intensity while the intensity of the cytoplasmic staining remained constant.

**Figure 6:**
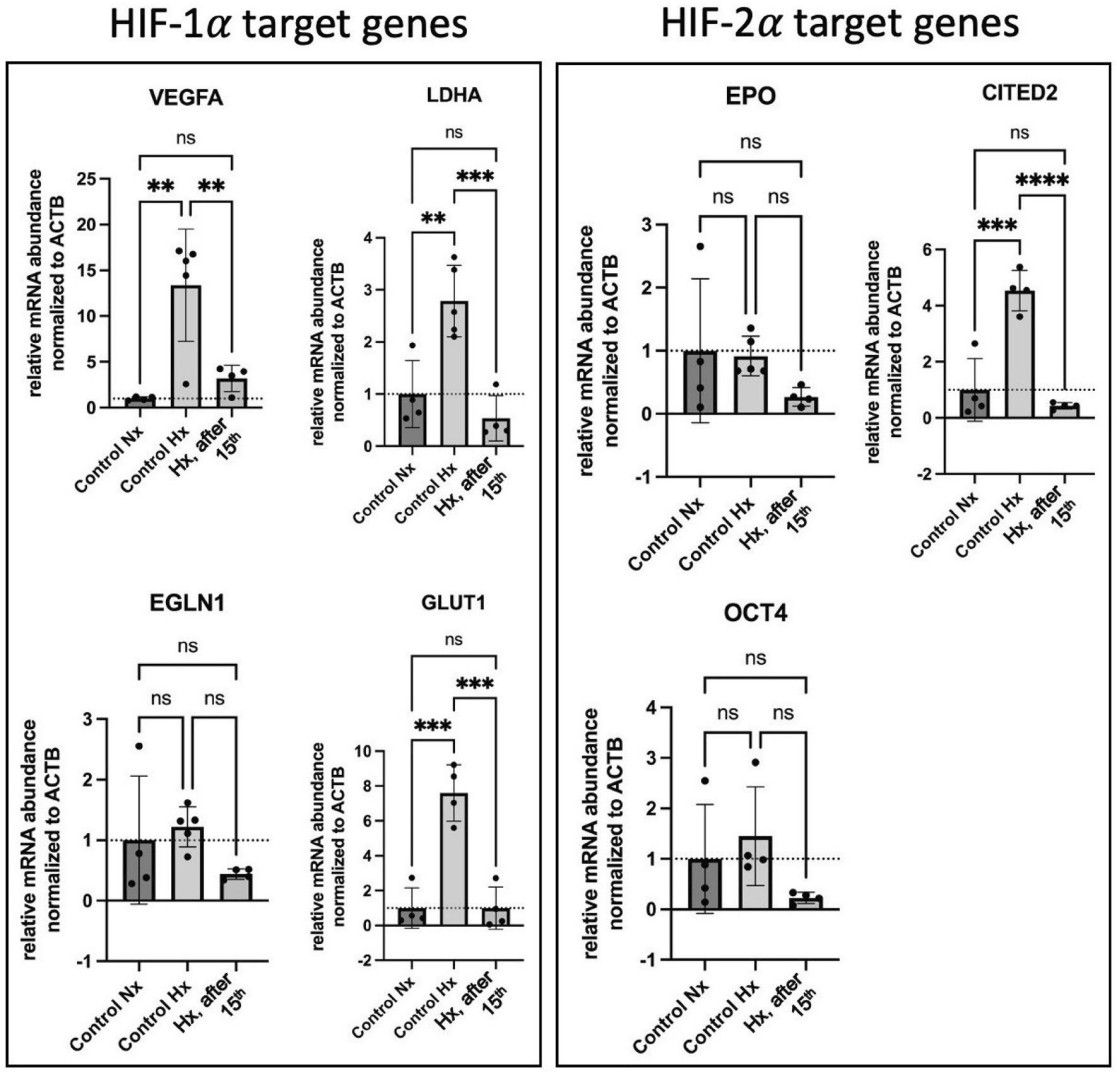
Hypoxia-induced expression of HIF1α and HIF2α-dependent genes. Analysis was performed after 75 min of constant 1g condition (control Hx) and after 75 min of hypoxia during 15 parabolic flight maneuvers (Hx, after 15th) compared to in-flight normoxic control (control Nx). Hypoxia-induced expression of all investigated genes was suppressed by a series of 15 parabola maneuvers compared to hypoxia-induced expression under constant 1g conditions. Only VEGFA was by trend more strongly expressed under hypoxia with 15 parabola maneuvers compared to normoxia. All examined genes showed a higher expression under hypoxia at a constant 1g. Data are shown as Means ± SD of 4–5 independent experiments. * = *P* < 0.05, ** = *P* < 0.01, **** = *P* < 0.0001. VEGFA: vascular endothelial growth factor A, LDHA: Lactate Dehydrogenase A, GLUT1: glucose transporter type 1, EGLN1: Egl-9 Family Hypoxia Inducible Factor 1 (= PHD2), EPO: erythropoietin, CITED2: CBP/p300 interacting transactivator with ED-rich tail 2, OCT4: POU class 5 homeobox transcription factor.

Equally important, RNA was harvested from cells cultured in normoxic devices (version 2) before induction of hypoxia and the 1st parabola, from those after induction of hypoxia and the 15th parabola, and from those exposed to hypoxia for 88 min in the ground lab in the same version 2 devices, followed by analysis for the expression of HIF1α and HIF2α-dependent genes. Expression of HIF1α-dependent genes, namely vascular endothelial growth factor A (VEGFA), Lactate Dehydrogenase A (LDHA), and glucose transporter type 1 (GLUT1) was suppressed by 4.19, 5.19, and 7.64 folds, respectively, by a series of 15 parabolas as compared to the expression under constant 1g hypoxic conditions (Fig. 6). Only VEGF was by trend more strongly expressed under hypoxia with 15 parabola maneuvers as compared to normoxia. Interestingly, this finding agrees with the well-known weightlessness-induced exacerbation of hypoxia symptoms such as visual disturbances or cerebral edema. Regarding expression of the HIF2α-dependent genes investigated, CBP/p300 interacting transactivator with ED-rich tail 2 (CITED2) was 10.5 times less expressed after the 15 parabolas as compared to the expression under constant 1g hypoxic conditions. All examined genes showed a higher expression under hypoxia at a constant 1g compared to hypoxia during 15 parabolas. This suggests that the cellular response to hypoxia is impaired by parabolic flight maneuvers.

### Myosin motors are required for proper nuclear translocation of HIF1α

**Figure 7:**
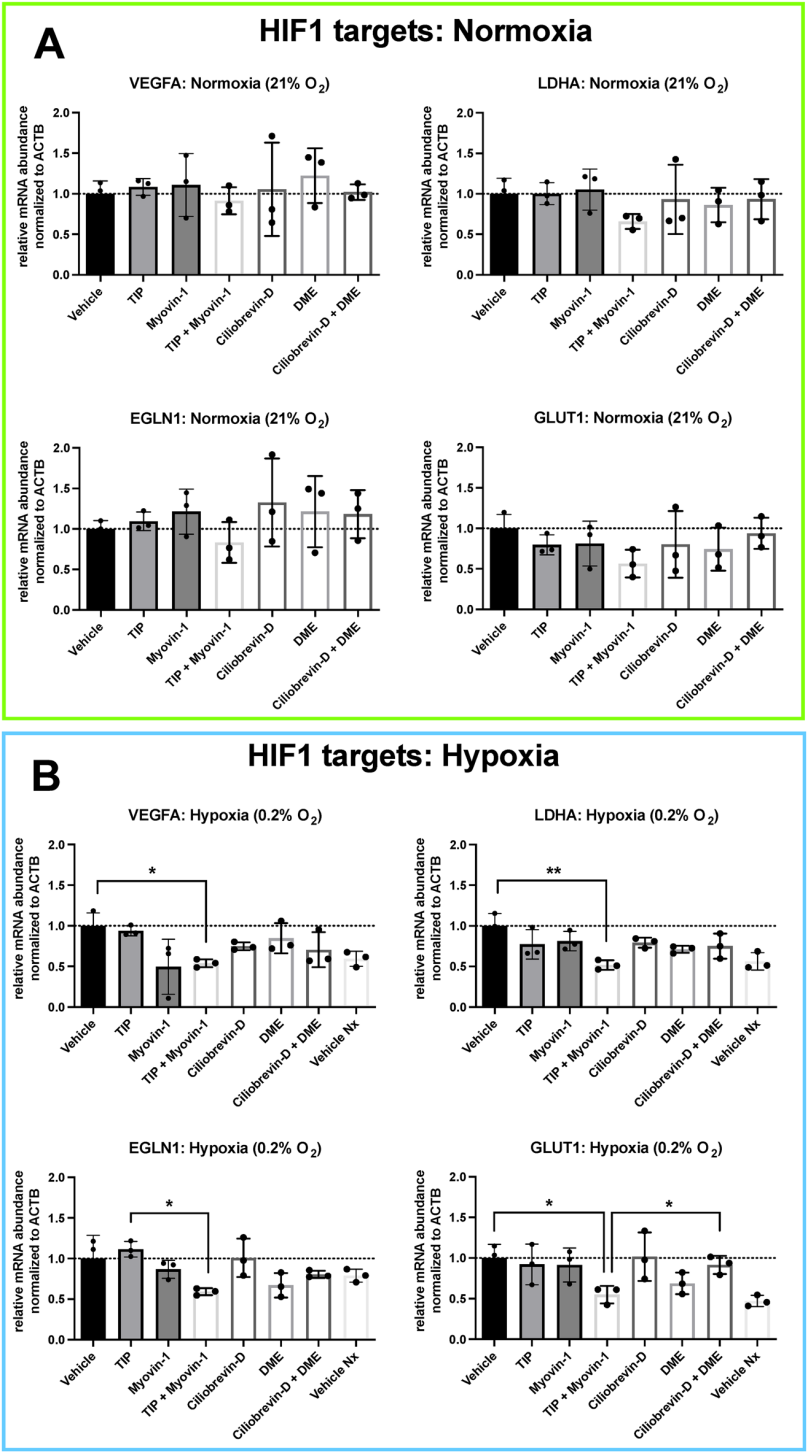

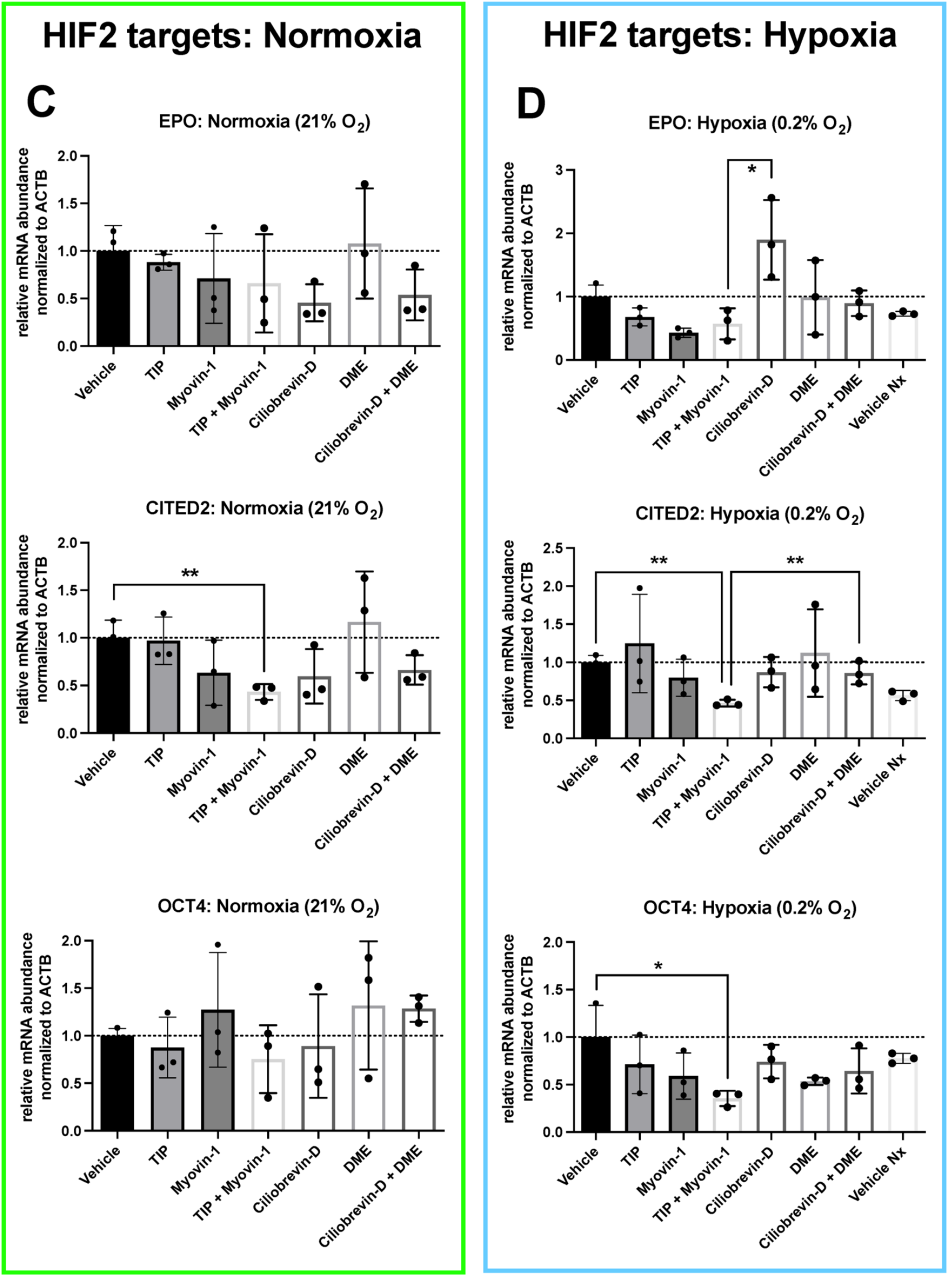
Cellular response to hypoxia while inhibiting cytoskeleton motor proteins. Panels (**A**) and (**B**) show the HIF1α-dependent target genes investigated under normoxia and under hypoxia. Panels (**C**) and (**D**) show the same for the HIF2α target genes. Inhibiting cytoskeletal-dependent intracellular transport during hypoxia with a combination of myosin V and myosin VI inhibitors suppressed the expression of all investigated genes as compared to vehicle control (DMSO-treated). A single treatment with either myosin V or VI inhibitors or with inhibitors of dynein or kinesin, again alone or in combination, had no significant effect. In the case of the HIF2α-dependent target genes, a similar expression pattern was found under hypoxia, however, only for two of the three genes examined. For B and D, Vehicle Nx (normoxic) control is significantly different from Vehicle (hypoxia) for VEGFA, LDHA, GLUT1, and CITED2, as well as non-significantly different from TIP + Myovin-1 for all markers. For the abbreviations of the genes see Fig. 6 legend. Data are shown as Means ± SD of 3 independent experiments per condition. * = *P* < 0.05, ** = *P* < 0.01. The inhibitors are TIP: 10 µM 2,4,6-triiodophenol (specific Myosin VI inhibitor), 50 µM Myovin1 (specific Myosin V inhibitor), 20 µM Ciliobrevin D (specific Dynein inhibitor) or 1 µM dimethylenastron (specific Kinesin inhibitor).

**Figure 8:**
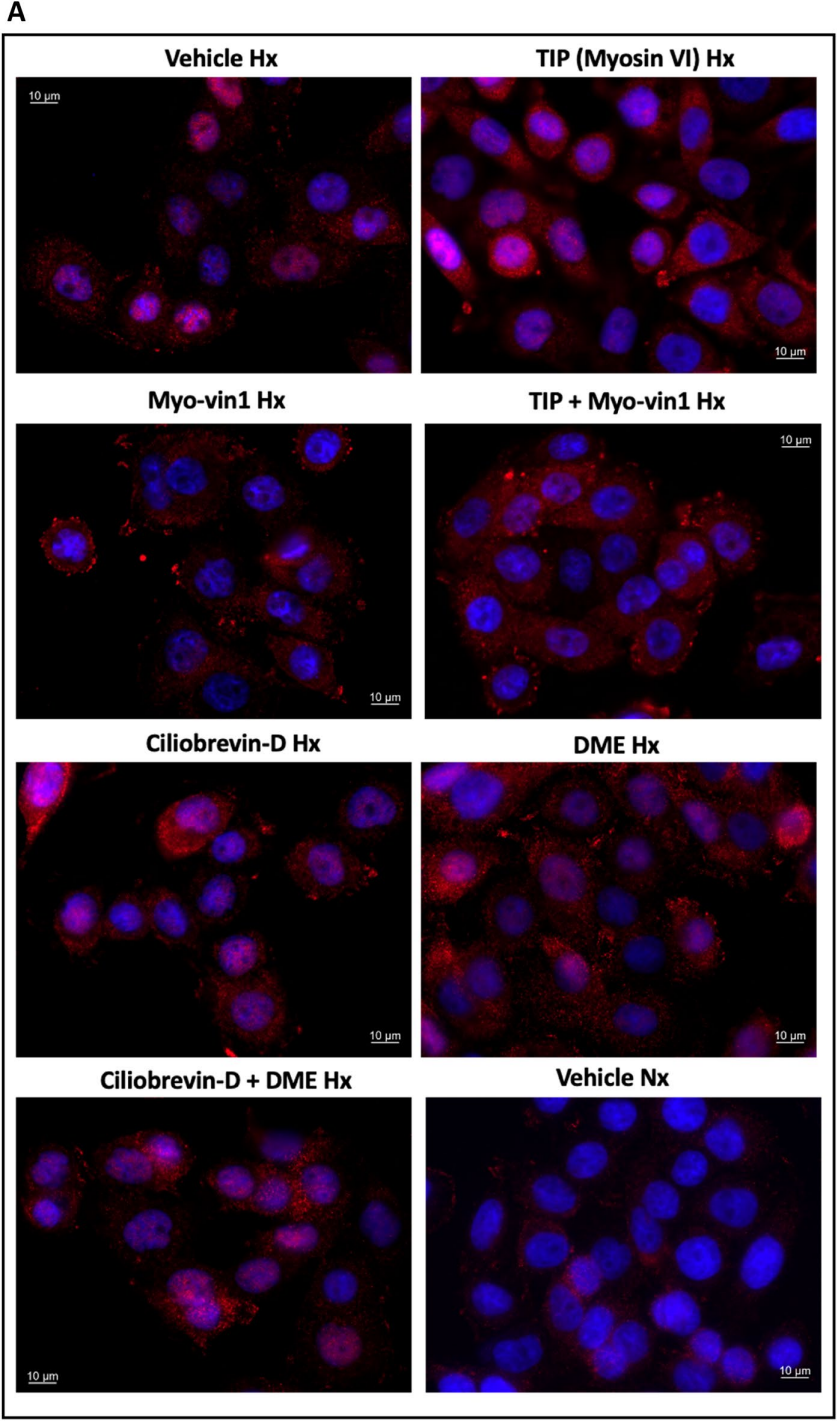

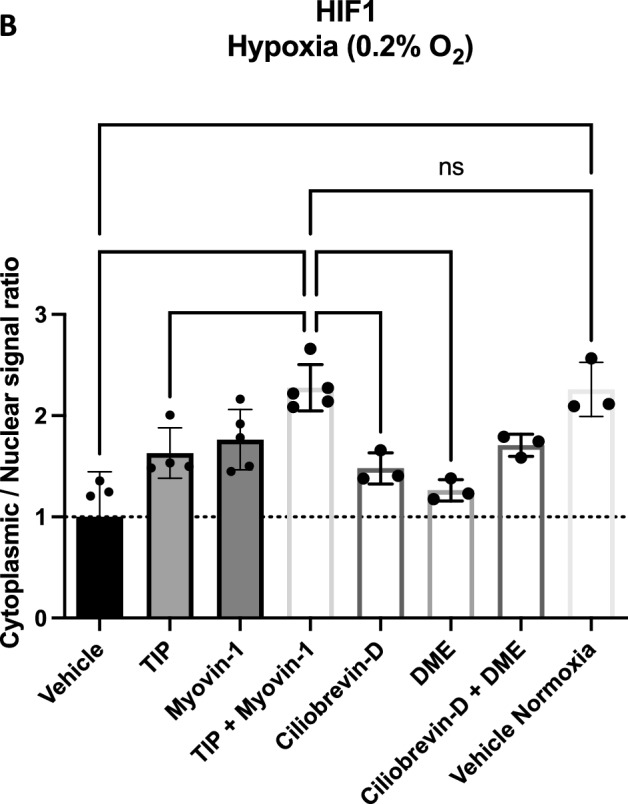
The ratio of the cytoplasmatic to nuclear HIF1α staining intensity under hypoxia combined with cellular motor protein inhibitions vs. normoxic vehicle-treated control. Panel (**A**) shows representative pictures of HIF1α immunostaining (red) versus DAPI counterstain (blue) with the different cellular motor protein inhibitors. Panel (**B**) shows the quantification of the ratio of the cytoplasmatic to nuclear HIF1α staining intensity obtained from these experiments. The ratio was by tendency increased by any inhibition of the cytoskeleton-dependent transport. However, only a combination of Myosin V and VI inhibition resulted in a significant increase in this ratio as compared to vehicle treatment (DMSO) or to most of the other groups under hypoxia, and to a similar level to normoxic control. For the meanings of the abbreviations see Fig. 7. Data are shown as Means ± SD of 3–5 independent experiments. * = *P* < 0.05, ** = *P* < 0.01, **** = *P* < 0.0001.

Based on our results and the previous findings that the cytoskeleton might be involved in the cellular hypoxic response^23^, we attempted to simulate the alleged inhibitory effect of parabolic flights on the cellular hypoxic response by inhibiting the cytoskeletal-dependent intracellular transport during hypoxia without exposing the cells to any parabola. The results of these experiments are summarized in Figs. 7 and 8. Compared to control conditions (vehicle treatment), hypoxia-induced expression of all investigated HIF1α target genes was suppressed with a combination of myosin V and myosin VI inhibitors in contrast to the single inhibition of either myosin or single and combined inhibition of dynein or kinesin (Fig. 7), showing an equivalent expression pattern to normoxic exposure. Regarding HIF2α-dependent target genes, a similar pattern was found under hypoxia for two of the three genes examined. In line with these findings, the cytoplasmic to nuclear staining ratio for HIF1α was significantly increased again only with concomitant inhibition of myosin V and VI as compared to vehicle treatment, single inhibitors, or treatment with single or combined inhibitors of the other cytoskeleton-dependent transporters, dynein or kinesin, under hypoxia, implying hindered nuclear translocation of HIF and restoring the pattern witnessed under control normoxic exposure (Fig. 8).

To explore the alleged interaction between HIF1α and myosin V and/or VI, we next performed an in-silico analysis, utilizing the STRING database (https://string-db.org/), to reveal any known or predicted protein–protein interactions. Although the ideal verification should be drawn from experiments, digging such a big database of physical or functional interactions is helpful to gain insight into which and how our proteins of interest may interact. STRING did not find direct interactions of HIF1α with either myosin V, myosin VI, kinesin, or dynein. However, as HIF1α interacts with heat shock protein 90 (HSP90) that in turn accelerates HIF1α stabilization^28,29^ and hence is essential for HIF1α activation in hypoxia^30^, we included this protein in our analysis. In this setting, STRING indeed predicts an interaction for HIF1α via HSP90AB1 (Hsp90 beta) and calmodulin1-3^31,32^ with all myosin V isoforms as well as with myosin VI. Regarding kinesin (or its subunits), no interaction with HIF1α was found in the presence or absence of HSP90 or calmodulins. Thus, this observation confirms the aforementioned results that inhibiting the motor proteins myosin V and VI, but not kinesin, impacted HIF1a nuclear translocation. In contrast, dynein was predicted to interact with HIF1α via HSP90 while when we inhibited dynein HIF1α translocation seemed unaffected. This analysis suggests both myosin V and VI motors are required for HIF1*α* translocation to the nucleus via interacting with HSP90 and calmodulins.

## Discussion

We show that the cellular response to acute hypoxia (< 90 min) is suppressed by altered gravity (1–1.8–0–1.8–1g, 15 consecutive parabolas consisting each of 20 s at 1.8 g and 22 s at 0g phases) and that this could be due to impaired nuclear translocation of HIF1α. This observation might be attributed to disturbed cytoskeletal transport during SGCs, as evidenced by the altered phalloidin-stained cytoskeleton structure. The latter is in line with our previous observation during the 1st Swiss parabolic flight campaign that, after the first parabola, numerous MDA-MB-468 cells showed single to few protrusions or lamellipodia, which after the 15th parabola were seen in almost all cells along their entire cellular circumference^9^. Moreover, this is in line with our previous findings that HIF1α-driven cellular hypoxic response could be cytoskeleton dependent^23^ and with the fact that proteins, RNA, vesicles, etc. are transported within the cell along the cytoskeleton^24^. Disturbance of intracellular transport due to SGCs finds its physical basis in the different mass densities and, thus, inertia between proteins and cytoplasm. Consequently, we could show that actin-dependent intracellular transport inhibitors also suppressed the cellular hypoxic response.

Macromolecules like vesicles, RNA, and proteins do not just randomly diffuse around within cells to reach their intracellular target location. Instead, they are transported along the cytoskeleton either on microtubules or on actin filaments with the aid of different molecular motor proteins, such as kinesin, dynein, and myosins, mainly V, VI^24^. This is necessary to rapidly deliver macromolecules to specific intracellular locations and to avoid diffusion limitations due to mechanical hindrance by the cytoskeletal network. Finally, some cells are too large (e.g., neurons with long axons, oocytes, etc.) to even theoretically rely on diffusion for the distribution of macromolecules (http://book.bionumbers.org/what-are-the-time-scales-for-diffusion-in-cells/). In general, cargos are attached to 1–3 (up to 5) motors that are “walking” along their track. The type of motor attached to the cargo determines the direction of transport, either to or away from the nucleus^24^. Sometimes a cargo can be attached to even two different types of motors that may then allow a change in the direction of transport or even a change of the type of track (microtubules vs. actin)^33^. The transport speed and probability of separation from the track are functions of cargo weight and the number of motors. Myosin V^34^ and kinesins^35^ have been shown to reduce their processivity with the load. Moreover, in the absence of ATP, dynein steps processively along microtubules under an external load, with less force required for minus-end- than for plus-end-directed movement^36^. These reports are important regarding our study because SCGs will pull on the load due to the inertia of the proteins relative to the cytoplasm, as cytoplasm and proteins have significantly different mass densities. Specifically, the density of the cytoplasm is approximately 1 g/cm^3^ since it consists mainly of water (80–85%). The average protein density had been assumed to be equal to 1.35 g/cm^3^ independently of the protein’s nature or its molecular weight. However, it was later shown that average protein density is molecular weight dependent^37^. Lower molecular weight proteins are associated with a higher density (up to 1.52 g/cm^3^) whereas proteins with higher molecular weights (such as HIF1α with a molecular weight of about 120kD^38^) have a lower density approximating asymptotically an average of 1.41 g/cm^3^^37^. Of note, macromolecules or other cargo attached via motor molecules to either actin filaments or microtubules stick out into the cytoplasm like branches from a tree trunk. Thus, the load (at the end of the branch) of any molecular motor will be exposed to the whole density difference between the cytoplasm and the load itself. Consequently, the inertia force that is suddenly increased at the beginning of a parabola or suddenly released in transition to 0 g and vice versa instantly increases the load on the connection between track, motor molecule, and cargo and, thus, the probability of separation from the track.

Based on our observation that SGCs impair the cellular hypoxic response and considering our hypothesis that this might be due to disturbed cytoskeleton-dependent nuclear translocation of the HIFs, we exposed MDA-MB-468 breast cancer cells to 60 min of 0.2% oxygen concentration while inhibiting different cytoskeleton-dependent motor molecules. Indeed, we found the expression of HIF1α downstream target genes suppressed by a combination of myosin V and VI inhibitors, whereas inhibition of myosin V or myosin VI alone as well as inhibition of kinesin or dynein alone or in combination had no effect. Regarding HIF2α target genes, there was no change with motor proteins inhibition, which might be attributed to the delayed response of HIF2α^39^. STRING platform predicts an interaction for HIF1α via HSP90AB1 (Hsp90 beta) and calmodulin1-3^31,32^ with all myosin V isoforms as well as with myosin VI but not kinesin. We also tested a previously published (indirect) interaction of HIF1α with dynein^40^ postulating a direct interaction between (hypoxia-stimulated) HIF1α and BIC-D1, the latter connecting the HIF1a-BIC-D1 complex to dynein and facilitated HIF1α nuclear import through nuclear pores with the aid of RAN-Bp2. However, this interaction between HIF1α, BIC-D1, and dynein was not indicated by STRING, but it predicted a connection of HIF1α to Ran-BP2 and BIC-D1 with dynein. Another report^41^ shows that microtubule-targeting drug (MTD) treatment impaired HIF1α nuclear translocation, which suppressed HIF1α transcriptional activity. Interestingly, Carbonaro et al. observed additionally that the microtubule-dependent HIF1α regulation was absent in renal cell carcinoma cells although these cells have normal HIF1α-dependent transcriptional activity^41^. This might allow the conclusion that the reported microtubule/dynein-dependent nuclear transfer of HIF1α is not imperatively the only mechanism for eukaryotic cells to get HIF1α into the nucleus to form functional transcription complexes necessary for the expression of oxygen-dependent regulated genes. In this context, it is worth mentioning that myosin VI is an early marker of tumorigenesis, aggressiveness, and invasion since it is dramatically up-regulated in various cancer cells^42,43^ including RCC^44^ and maybe also breast cancer cells^45^. In our study, we used breast cancer cells (MDA- MB-468) in which the expression of functional myosin V has been previously demonstrated^46^.

Regarding the other important proteins in the network predicted by STRING, it is worth mentioning that calmodulin regulates the binding of the HSP90 to actin filaments. At low intracellular calcium concentrations, HSP90 is able to bind to actin filaments whereas HSP90 complexed with Ca^2+^-calmodulin is unable to bind to F-actin^32,47^ and the binding of HSP90 to calmodulin requires calcium^31^. Since hypoxia is known to increase the intracellular Ca^2+^ concentration^48^, this will likely, first, trigger the release of HSP90 from actin filaments and, secondly, facilitate the binding of calmodulin to HSP90. In addition, increased intracellular Ca^2+^ concentrations reduce sliding velocity and binding of myosin V to actin^49^ but are required to activate the motor to bind cargo and to allow movement of myosin VI with the attached cargo towards the cell center once the intracellular calcium returns to low values^45^. Reduced activity of myosin V and VI might be because these proteins are unusual concerning calmodulin binding that occurs at low intracellular calcium concentrations^50,51^. Interaction of HIF1α and HSP90 stabilizes HIF1α, thus, disruption of this interaction, e.g., by inertia forces (MW of HSP90 = 90kD, HIF1α = 120kD or different densities and much higher density compared to cytoplasm) may accelerate HIF1α degradation. During a short duration of hypoxia, the hypoxic response was reported to increase when calcium is inhibited^52^. However, others found in CALM^−/−^ cells that HIF1α-dependent transcriptional activity was decreased by 40% and increased by ionomycin treatment that rises calcium^53^. This could add up in the context of our experiments since the putative release of the HSP90-HIF1α complex from actin filaments would be triggered by intracellular calcium increases, thus, mobilizing HIF1α for subsequent transport to the nucleus. Another interesting observation is that HSP90 inhibitors at low concentrations can activate the HIF1α response whereas higher doses inhibited HIF1α activity^54^. It could be speculated that low inhibitor doses result in more unbound HIF1α that will mainly be transported to the nucleus if enough transport molecules (dyneins) are available. At higher doses, too much HIF1α could be released at once thereby occupying all available dyneins for transport and subsequently, the surplus of released HIF1α could be degraded. Of note, it has been recently shown that the Calcium release-activated channel regulator 2A (CRACR2a) protein binds to the dynein-cargo complex and that physiological elevations of intracellular calcium (i.e., from 10 nM to 2 µM) increased the processivity of dynein–dynactin–CRACR2a complexes on microtubules, indicating that elevated intracellular calcium concentrations can speed up nucleopetal dynein-dependent cargo transport^55^. This also matches the notion that increased intracellular calcium concentration augments the hypoxic response of HIF1α.

We used a novel miniaturized experimental cell culture system that allows setting cells to near anoxia very rapidly. Due to operational restrictions by the aircraft operator, we were not allowed to keep our system running for a period of 45 min after take-off during which the cells cooled down. This could have negatively influenced our experiments since this cold stress might have altered HSP90 expression^56,57^. Another shortcoming of the parabolic flight lies in the fact that the number of parabolas and thus the time of SGCs is limited to about 90 min. Thus, it would be interesting to have a platform allowing to provide SGCs for a few hours. We are currently working on a new platform that will overcome both methodological constraints.

In conclusion, we report for the first time that the cellular response to hypoxia is impaired by altered gravity may be due to disruption of HIF1α interaction via HSP90 and myosin V and VI with actin. This interaction appears to be required for proper nuclear translocation of HIF1α. We hypothesize that HIF1α, when complexed with HSP90, binds to actin filaments that act as an intermediate store for HIF1α. SCGs will disrupt this binding leading to impaired transport to the nucleus and perhaps increased degradation of HIF1α (since more free-floating HIF1α is present). Myosin V and VI inhibitors block movement and detachment of myosins from actin^58,59^. Consequently, more myosin heads stay bound to actin filaments which may introduce a steric inhibition for HSP90-HIF1α binding to actin. This could also explain why myosin V and VI inhibitors alone have no significant effect in contrast to the combined inhibition of these myosins. Moreover, dynein inhibition did not affect HIF1α-target genes expression after 60 min. However, MTDs were reported to exert such an effect when measured after 4 h or more of hypoxia. Interestingly, this 4 h of MTD treatment under hypoxia did not affect total HIF protein levels, while it impacted the intracellular distribution of HIF-1α by impairing its nuclear accumulation^60^. This matches our hypothesis that actin filaments could be an intermediate store for HIF1α that is not affected by MTD treatment. Our study demonstrates at the molecular level that microgravity and changing gravity conditions interact with the HIF system. We are convinced that our observation will pave the way to better solve the clinical challenges of combining hypoxia and changing gravity in EVAs or military aviation.

### Supplementary Information

Supplementary Information.

## Data Availability

All data generated or analyzed during this study are included in this article and the supplementary material file.

## Abbreviations

HIF: Hypoxia-inducible factor
SGC: Sudden gravity changes
EVAs: Extravehicular activities
AMS: Acute mountain sickness
VIIP-Syndrome: Visual impairment/Intracranial pressure syndrome
HRE: Hypoxia response element
VEGF: Vascular endothelial growth factor
TIP: 2,4,6-Triiodophenol
DME: Dimethylenastron
SPFC: Swiss parabolic flight campaign
LDHA: Lactate dehydrogenase A
EGLN1: Egl-9 family hypoxia inducible factor 1
GLT1: Glucose transporter type 1
EPO: Erythropoietin
CITED2: CBP/p300 interacting transactivator with ED-rich tail 2
OCT4: POU class 5 homeobox transcription factor
ACTB: β-Actin
MEMS: Micro-electro-mechanical system
HSP90: Heat shock protein 90
MTD: Microtubule-targeting drug
CALM: Calmodulin

## References

[1] Gotoh TM (2004). Acute hemodynamic responses in the head during microgravity induced by free drop in anesthetized rats. Am. J. Physiol. Regul. Integr. Comp. Physiol..

[2] Imray C, Wright A, Subudhi A, Roach R (2010). Acute mountain sickness: Pathophysiology, prevention, and treatment. Prog. Cardiovasc. Dis..

[3] Simonson TS (2015). Altitude adaptation: A glimpse through various lenses. High Alt. Med. Biol..

[4] Hormeño-Holgado AJ, Clemente-Suárez VJ (2019). Effect of different combat jet manoeuvres in the psychophysiological response of professional pilots. Physiol. Behav..

[5] Clemente-Suárez VJ, Robles-Pérez JJ (2013). Mechanical, physical, and physiological analysis of symmetrical and asymmetrical combat. J. Strength Cond. Res..

[6] Petrassi FA, Hodkinson PD, Walters PL, Gaydos SJ (2012). Hypoxic hypoxia at moderate altitudes: Review of the state of the science. Aviat. Space Environ. Med..

[7] Vahlensieck C, Thiel CS, Zhang Y, Huge A, Ullrich O (2021). Gravitational force-induced 3D chromosomal conformational changes are associated with rapid transcriptional response in human T cells. Int. J. Mol. Sci..

[8] Vahlensieck C (2021). Rapid transient transcriptional adaptation to hypergravity in jurkat T cells revealed by comparative analysis of microarray and RNA-Seq data. Int. J. Mol. Sci..

[9] Vogel J (2019). Expression of hypoxia-inducible factor 1alpha (HIF-1alpha) and genes of related pathways in altered gravity. Int. J. Mol. Sci..

[10] Moser D (2019). Cells’ flow and immune cell priming under alternating g-forces in parabolic flight. Sci. Rep..

[11] Kaufmann I (2009). Parabolic flight primes cytotoxic capabilities of polymorphonuclear leucocytes in humans. Eur. J. Clin. Invest..

[12] Kaufmann I (2011). Adenosine A2(A) receptor modulates the oxidative stress response of primed polymorphonuclear leukocytes after parabolic flight. Hum. Immunol..

[13] Staub K (2020). Hemoglobin concentration of young men at residential altitudes between 200 and 2000 m mirrors Switzerland's topography. Blood.

[14] Jewell UR (2001). Induction of HIF-1alpha in response to hypoxia is instantaneous. FASEB J.

[15] Hong WX (2014). The role of hypoxia-inducible factor in wound healing. Adv. Wound Care (New Rochelle).

[16] Imtiyaz HZ, Simon MC (2010). Hypoxia-inducible factors as essential regulators of inflammation. Curr. Top. Microbiol. Immunol..

[17] Shweiki D, Itin A, Soffer D, Keshet E (1992). Vascular endothelial growth factor induced by hypoxia may mediate hypoxia-initiated angiogenesis. Nature.

[18] Zachary I (2003). VEGF signalling: Integration and multi-tasking in endothelial cell biology. Biochem. Soc. Trans..

[19] Connolly DT (1989). Tumor vascular permeability factor stimulates endothelial cell growth and angiogenesis. J. Clin. Invest..

[20] Meehan RT (1987). Immune suppression at high altitude. Ann. Emerg. Med..

[21] Klokker M, Kharazmi A, Galbo H, Bygbjerg I, Pedersen BK (1993). Influence of in vivo hypobaric hypoxia on function of lymphocytes, neutrocytes, natural killer cells, and cytokines. J. Appl. Physiol..

[22] Crucian B, Sams C (2009). Immune system dysregulation during spaceflight: Clinical risk for exploration-class missions. J. Leukoc. Biol..

[23] Antoniou X, Gassmann M, Ogunshola OO (2011). Cdk5 interacts with Hif-1alpha in neurons: A new hypoxic signalling mechanism?. Brain Res..

[24] Gross SP, Vershinin M, Shubeita GT (2007). Cargo transport: Two motors are sometimes better than one. Curr. Biol..

[25] Bicker A (2020). The role of myoglobin in epithelial cancers: Insights from transcriptomics. Int. J. Mol. Med..

[26] Borisov SM, Nuss G, Klimant I (2008). Red light-excitable oxygen sensing materials based on platinum(II) and palladium(II) benzoporphyrins. Anal. Chem..

[27] Jokic T (2012). Highly photostable near-infrared fluorescent pH indicators and sensors based on BF2-chelated tetraarylazadipyrromethene dyes. Anal. Chem..

[28] Gradin K (1996). Functional interference between hypoxia and dioxin signal transduction pathways: Competition for recruitment of the Arnt transcription factor. Mol. Cell Biol..

[29] Katschinski DM (2004). Interaction of the PAS B domain with HSP90 accelerates hypoxia-inducible factor-1alpha stabilization. Cell Physiol. Biochem..

[30] Minet E (1999). Hypoxia-induced activation of HIF-1: Role of HIF-1alpha-Hsp90 interaction. FEBS Lett..

[31] Minami Y, Kawasaki H, Suzuki K, Yahara I (1993). The calmodulin-binding domain of the mouse 90-kDa heat shock protein. J. Biol. Chem..

[32] Nishida E, Koyasu S, Sakai H, Yahara I (1986). Calmodulin-regulated binding of the 90-kDa heat shock protein to actin filaments. J. Biol. Chem..

[33] Arnold DB (2009). Actin and microtubule-based cytoskeletal cues direct polarized targeting of proteins in neurons. Sci. Signal.

[34] Veigel C, Schmitz S, Wang F, Sellers JR (2005). Load-dependent kinetics of myosin-V can explain its high processivity. Nat. Cell Biol..

[35] Bensel BM (2020). The mechanochemistry of the kinesin-2 KIF3AC heterodimer is related to strain-dependent kinetic properties of KIF3A and KIF3C. Proc. Natl. Acad. Sci. U. S. A..

[36] Gennerich A, Carter AP, Reck-Peterson SL, Vale RD (2007). Force-induced bidirectional stepping of cytoplasmic dynein. Cell.

[37] Fischer H, Polikarpov I, Craievich AF (2004). Average protein density is a molecular-weight-dependent function. Protein Sci..

[38] Wang GL, Semenza GL (1995). Purification and characterization of hypoxia-inducible factor 1. J. Biol. Chem..

[39] Jaśkiewicz M (2022). The transition from HIF-1 to HIF-2 during prolonged hypoxia results from reactivation of PHDs and HIF1A mRNA instability. Cell Mol. Biol. Lett..

[40] Lee HJ (2019). BICD1 mediates HIF1α nuclear translocation in mesenchymal stem cells during hypoxia adaptation. Cell Death Differ..

[41] Carbonaro M, Escuin D, O'Brate A, Thadani-Mulero M, Giannakakou P (2012). Microtubules regulate hypoxia-inducible factor-1α protein trafficking and activity: Implications for taxane therapy. J. Biol. Chem..

[42] Dunn TA (2006). A novel role of myosin VI in human prostate cancer. Am. J. Pathol..

[43] Su AI (2001). Molecular classification of human carcinomas by use of gene expression signatures. Cancer Res..

[44] Ronkainen H, Kauppila S, Hirvikoski P, Vaarala MH (2010). Evaluation of myosin VI, E-cadherin and beta-catenin immunostaining in renal cell carcinoma. J. Exp. Clin. Cancer Res..

[45] Batters C, Brack D, Ellrich H, Averbeck B, Veigel C (2016). Calcium can mobilize and activate myosin-VI. Proc. Natl. Acad. Sci. U. S. A..

[46] Zhang R (2011). Two-photon 3D FIONA of individual quantum dots in an aqueous environment. Nano Lett..

[47] Koyasu S (1986). Two mammalian heat shock proteins, HSP90 and HSP100, are actin-binding proteins. Proc. Natl. Acad. Sci. U. S. A..

[48] Arnould T, Michiels C, Alexandre I, Remacle J (1992). Effect of hypoxia upon intracellular calcium concentration of human endothelial cells. J. Cell Physiol..

[49] Nguyen H, Higuchi H (2005). Motility of myosin V regulated by the dissociation of single calmodulin. Nat Struct Mol Biol.

[50] Trybus KM (2007). Effect of calcium on calmodulin bound to the IQ motifs of myosin V. J. Biol. Chem..

[51] Trybus KM (2008). Myosin V from head to tail. Cell Mol. Life Sci..

[52] Berchner-Pfannschmidt U (2004). Chelation of cellular calcium modulates hypoxia-inducible gene expression through activation of hypoxia-inducible factor-1alpha. J. Biol. Chem..

[53] Mottet D (2003). Role of ERK and calcium in the hypoxia-induced activation of HIF-1. J. Cell Physiol..

[54] Ibrahim NO (2005). Induction of the hypoxia-inducible factor system by low levels of heat shock protein 90 inhibitors. Cancer Res..

[55] Wang Y (2019). CRACR2a is a calcium-activated dynein adaptor protein that regulates endocytic traffic. J. Cell Biol..

[56] Healy DA (2006). Heat shock-induced protection of renal proximal tubular epithelial cells from cold storage and rewarming injury. J. Am. Soc. Nephrol..

[57] Liu Y, Xue N, Zhang B, Lv H, Li S (2022). Cold stress induced liver injury of mice through activated NLRP3/Caspase-1/GSDMD pyroptosis signaling pathway. Biomolecules.

[58] Heissler SM (2012). Kinetic properties and small-molecule inhibition of human myosin-6. FEBS Lett..

[59] Islam K (2010). A myosin V inhibitor based on privileged chemical scaffolds. Angew. Chem. Int. Ed. Engl..

[60] Escuin D, Kline ER, Giannakakou P (2005). Both microtubule-stabilizing and microtubule-destabilizing drugs inhibit hypoxia-inducible factor-1alpha accumulation and activity by disrupting microtubule function. Cancer Res..

