# Supplementary material for: Expression of hypoxia-inducible genes is suppressed in altered gravity due to impaired nuclear HIF1α accumulation
Source: Sci Rep. 2023 Sep 4;13:14514. doi: 10.1038/s41598-023-41686-1 (PMC10477221; doi:10.1038/s41598-023-41686-1)
Supplement: Supplementary file 1 — Supplementary Information. [file 41598_2023_41686_MOESM1_ESM.docx]

**Expression of hypoxia-inducible genes is suppressed in altered gravity due to impaired nuclear HIF1α accumulation**

*Mostafa A. Aboouf, Cora S. Thiel, Sergey M. Borisov, Svantje Tauber, Eva Bönzli, Nelli Schetle, Oliver Ullrich, Max Gassmann, Johannes Vogel*

***Supplemental material***

***Cell culture devices***

The devices that have been developed and manufactured by J.V. have a dimension of 15x20x6 cm and are powered by twelve rechargeable AA-size NiMh-batteries (Eneloop, Panasonic) in 6S2P configuration. Each cell has a nominal capacity of 1900mAh. Both versions of cell culture devices are internally warmed using a newly developed heating element (Fig. S1). Both versions measure and keep a programmable temperature (in our case 37°C), measure linear and angular acceleration forces in all space directions as well as barometric pressure, and store all data on micro-SD-cards for offline analysis.


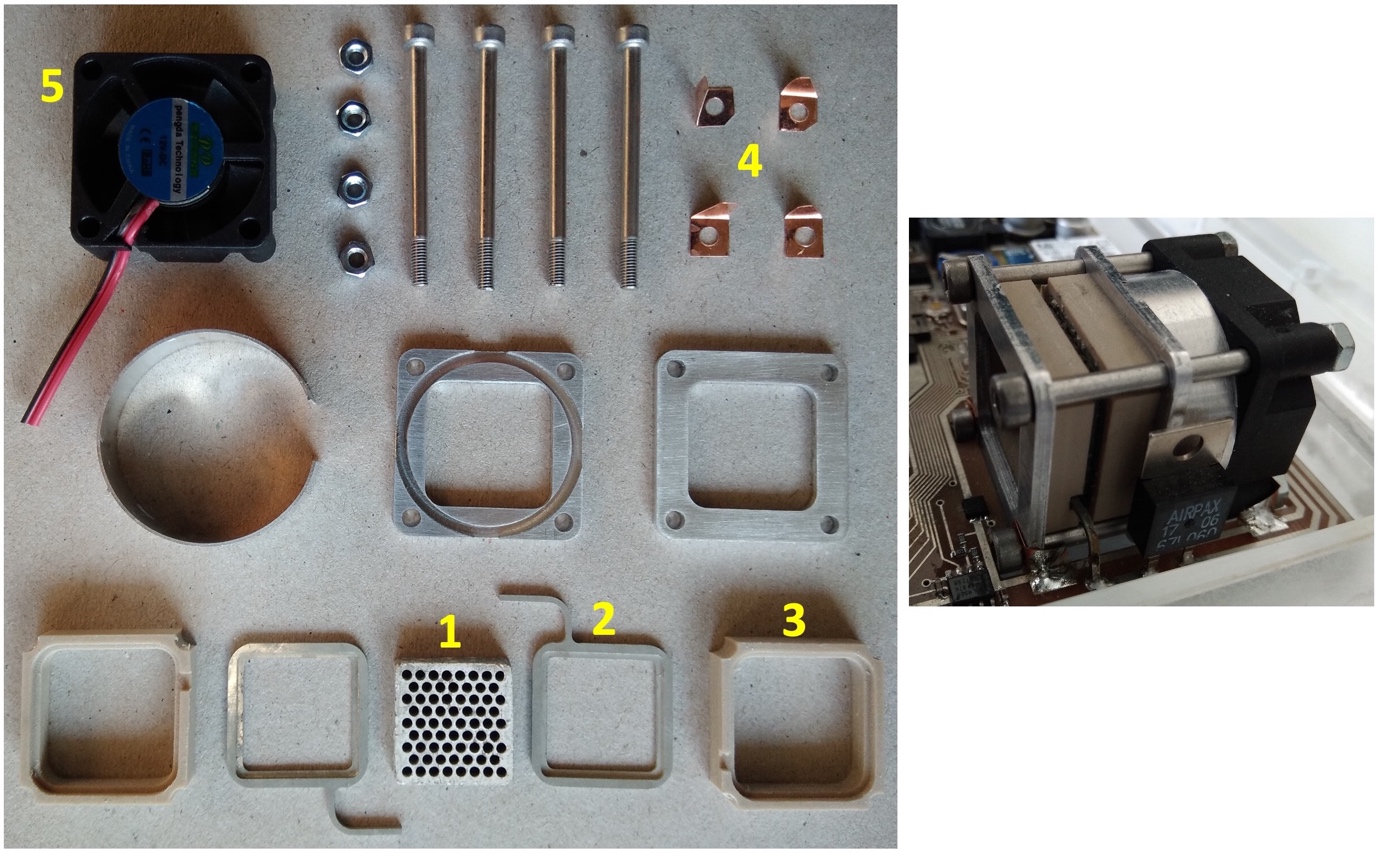


*Fig. S1.* Parts (left) and fully assembled and mounted (right) newly developed heating element. It is based on a PTC-ceramic (positive temperature coefficient). This ceramic increases its electrical resistance with temperature. Therefore, it cannot exceed a material-specific temperature, in this case, 140°C. The PTC-ceramic [1] is held between tinned brass contacts [2] and heat-resistive plastic (260°C, TECAPEEK [3]). Copper flaps [4] are used to solder the heater into its place on the PCB (right). The small, attached fan [5] blows the air through 61 holes in the ceramic to distribute the heat inside the device. The right panel also shows a bimetallic thermo-switch (AIRPAX 67L060) directly next to the heating element (see the text). The final size of the heating element is 3x3x4cm.

For safety reasons, the cell culture devices contain a bimetallic thermo-switch that cuts off the current supply to the heating element if the inside temperature would exceed 60°C (Fig. S1, right). We had to prove that the heater cannot melt or ignite the container that is made of plexiglass in case the bimetallic switch and the switching MOSFET fail. To this end, all control measures of the heating element were disabled, and the switched-on device was observed for 2h using an infrared camera. It was proven that even when all controls of the heating element are failing simultaneously the surface temperature of the cell culture device cannot exceed 59°C (Fig. S2).


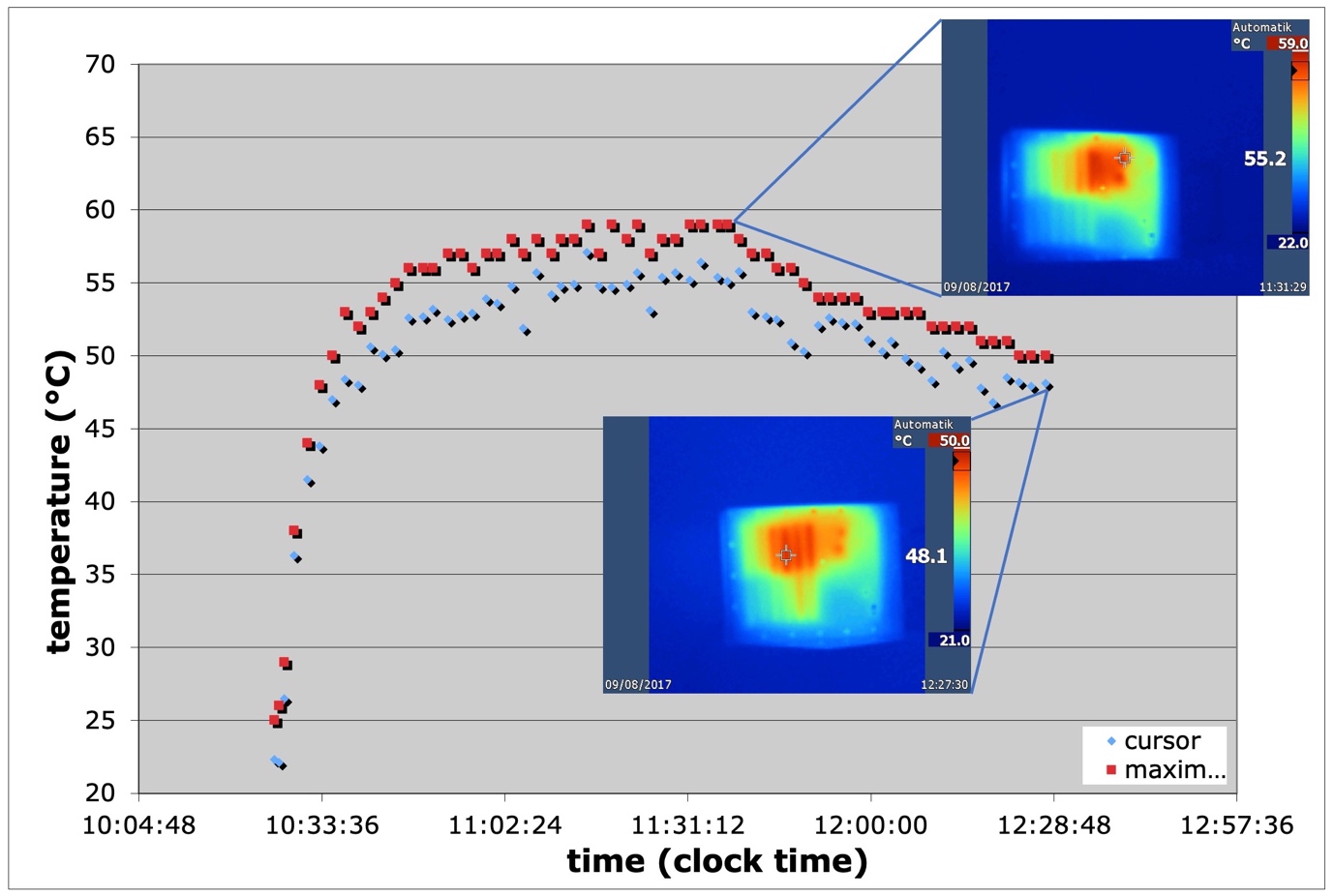


*Fig. S2.* To test that the device, does not melt, or ignite in case the heater control fails, the heater was set out of control by bridging the switching MOSFET and replacing the thermo-switch with a wire. The battery was fully charged, and the operating container was observed with an infrared camera for two hours. The red dots show the temperature of the hottest pixel of the respective image (red circle in the upper right inset) and the blue dots show the temperature of the curser (yellow circle in the lower inset). The surface temperature never exceeded 59°C. Note the decrease in temperature after about 80 min due to the exhaustion of the batteries.

Inside the cell culture devices, six separate cell cultures housed either in IBIDI-slides (µ-Slide I Luer, 0.8mm chamber height, [www.ibidi.com](http://www.ibidi.com)) in case of the version 1 devices and normoxic experiments or in oxygen-tight glass cell culture assemblies in case of the version 2 devices and hypoxic experiments (see Fig. 1 of the main manuscript) could be placed. Each cell culture is attached to two syringes for fluid exchange. Per cell culture device always six syringes are emptied into the cell cultures simultaneously by pushing one of its two levers. Details of the two device versions are depicted in figures S3-S8.

**1**

**5**


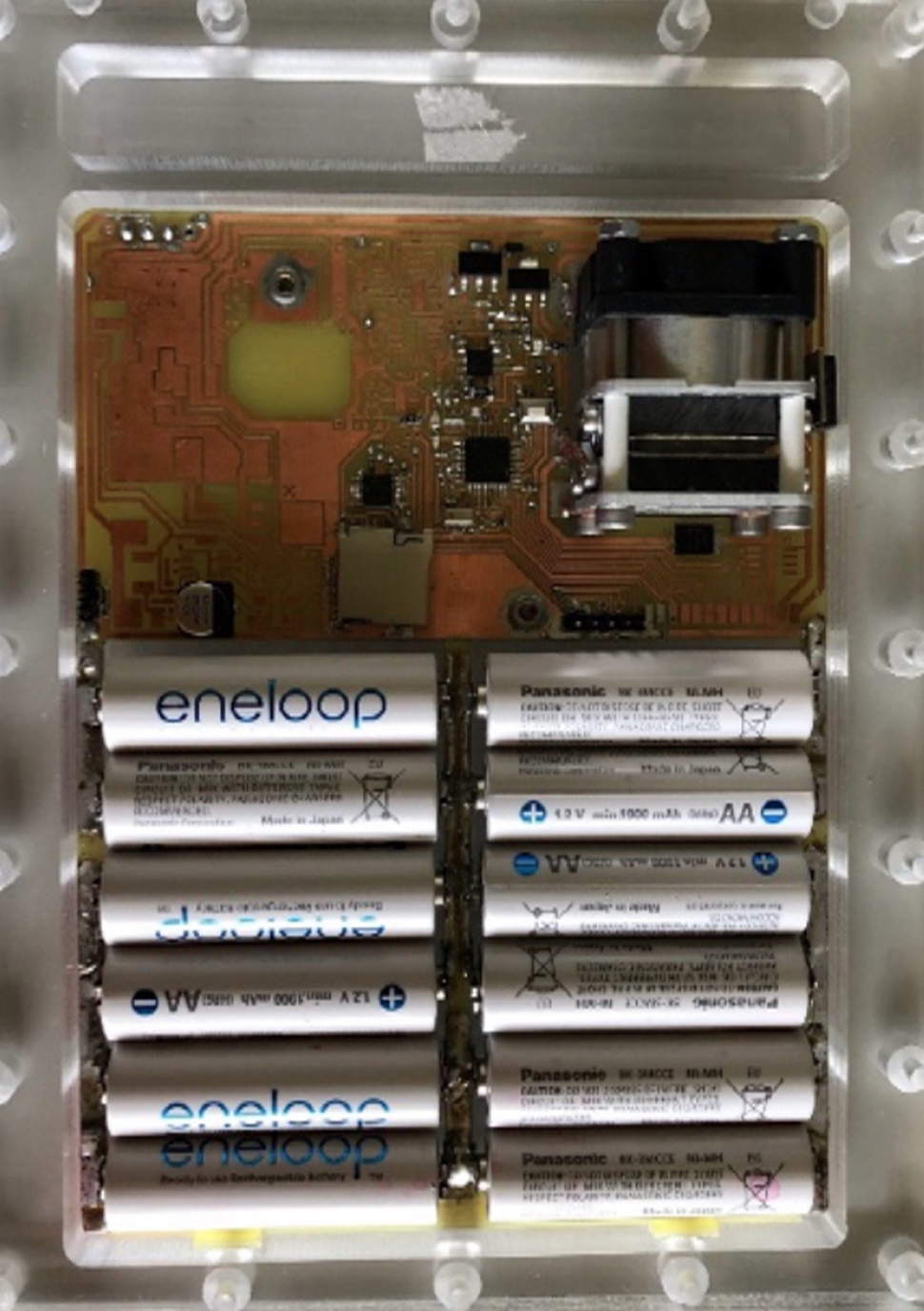


**2**

**3**

**4**

*Fig. S3.* Electronics of the cell culture device for normoxic experiments (version 1) that is controlled by the Atmega328P (Atmel, [1]). Linear acceleration and angular velocity as well as temperature are sensed by the MPU-6050 (InvenSense, [2]). Barometric pressure is measured by the MPL115A1 (NXP Semiconductors, [3]) that has an accuracy of ±10 hPa. The data sampling frequency to the micro-SD-card [4] is 5 Hz. In the lower right corner, the PTC-based heating element (detailed in Fig. S1) is visible [5].


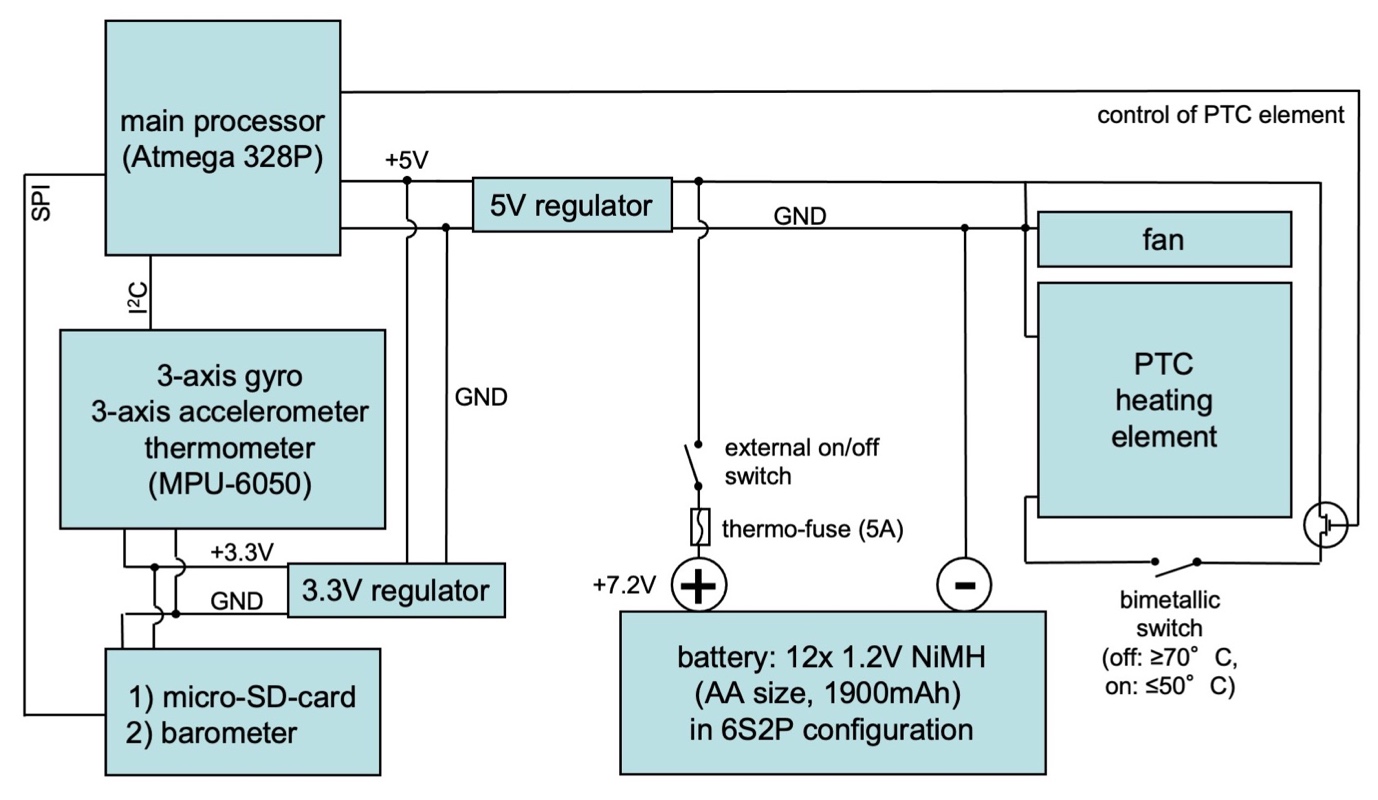


*Fig S4.* Electronic block diagram of the cell culture device used for normoxia experiments (shown in Fig. S3) as finally approved by the safety engineer of Novespace.


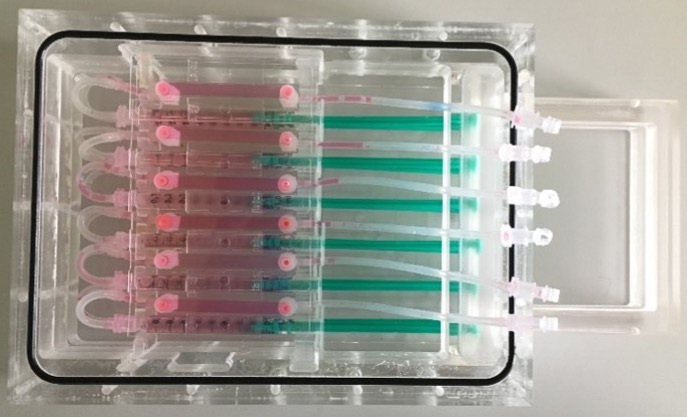


*Fig. S5.* Body of the normoxia cell culture device equipped with six IBIDI slides (IBIDI, Cat. No: 80196, channel height: 0.8mm), each of which attached to two syringes, both filled with 4% formalin solution to fix the cells. The fluid waste is directed into a waste compartment filled with napkins as soaking material.


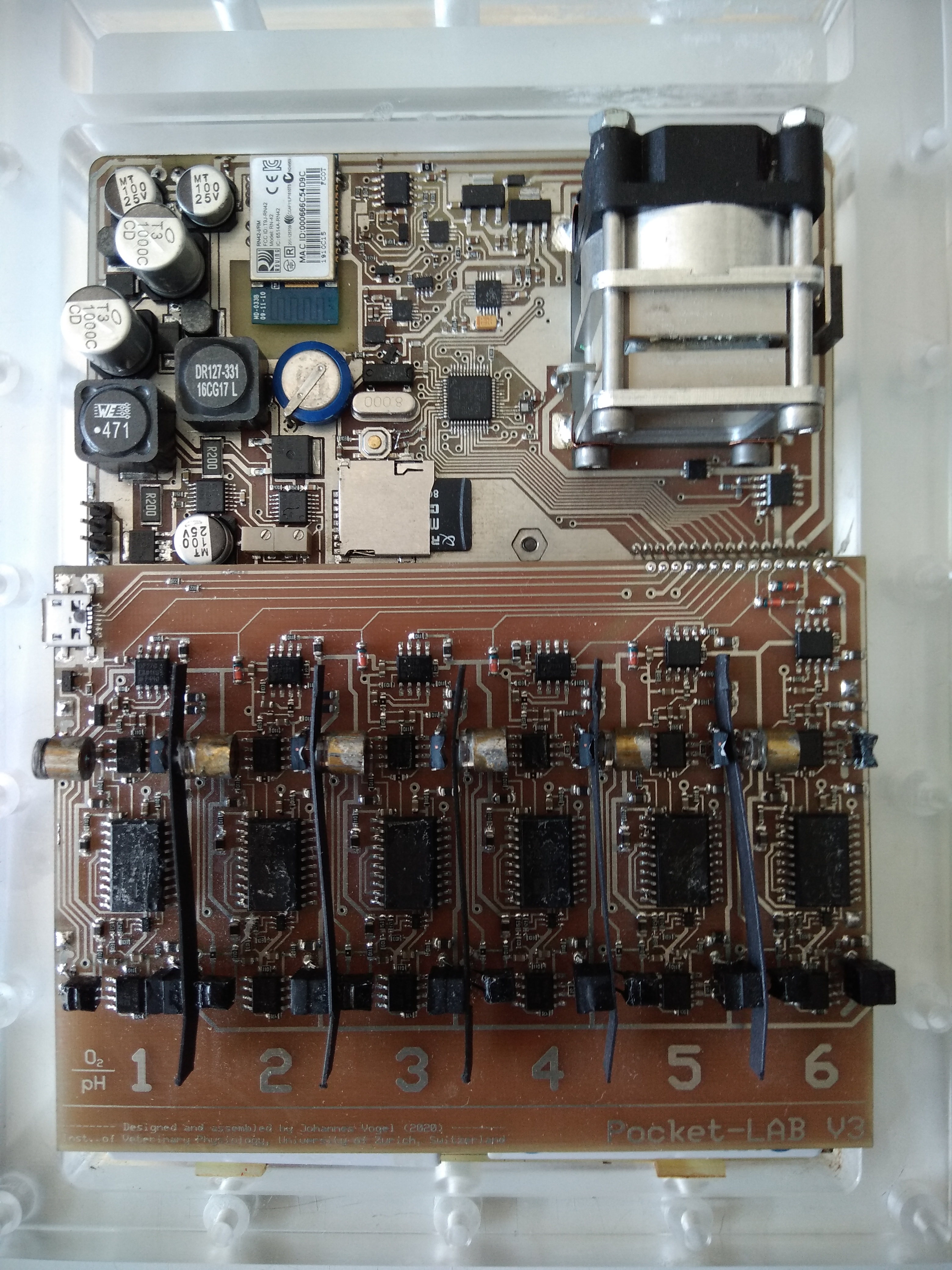


**1**

**3**

**2**

**4**

**11**

**5**

**6**

**7**

**8**

**9**

**100**

**B**

**A**


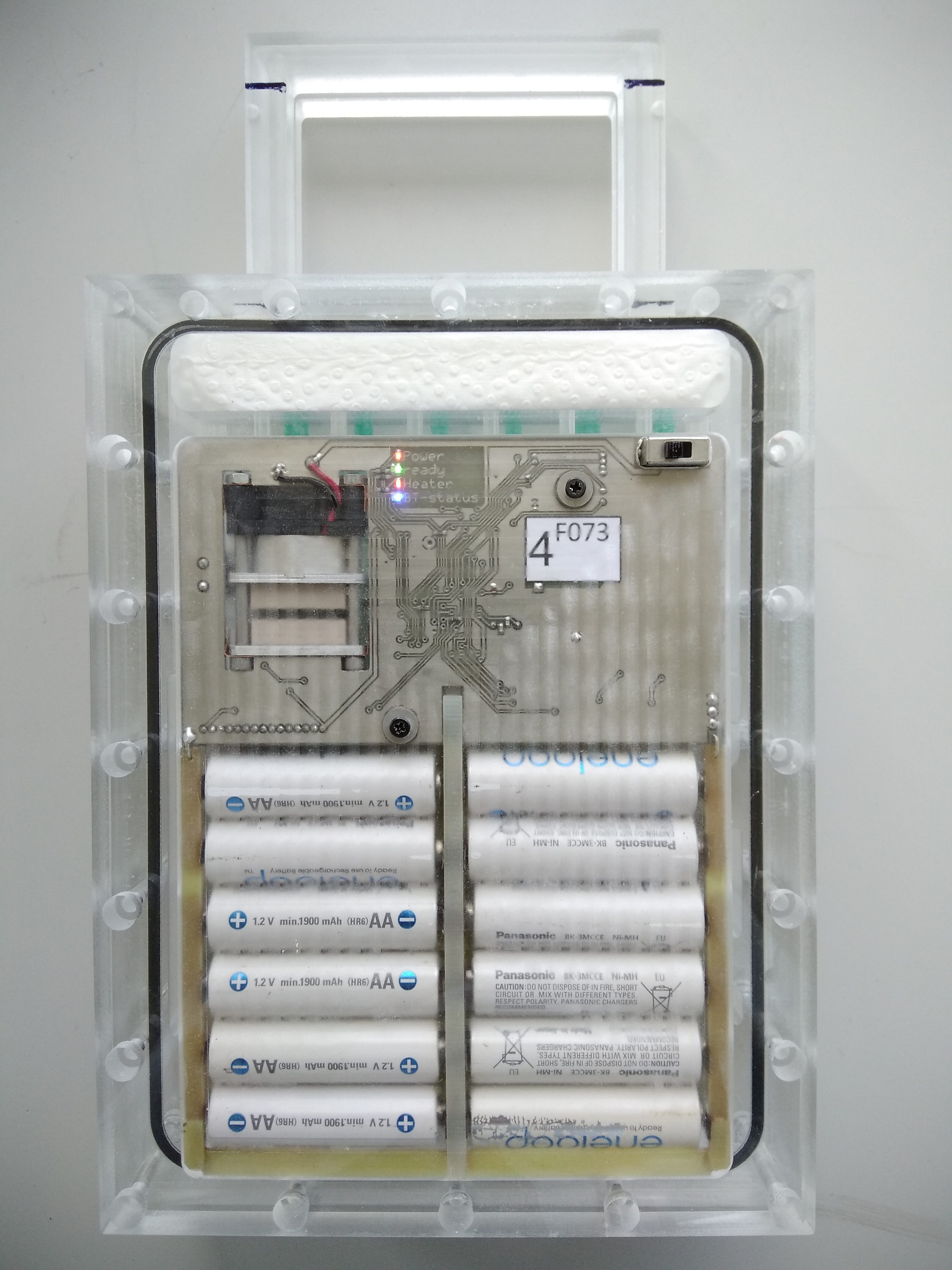


*Fig. S6.* Panel **A:** Electronics of the cell culture device for hypoxia experiments (version 2) that is controlled by the STM32F103C8 (STMicroelectronics, [1]). Linear acceleration and angular velocity as well as temperature are sensed by the MPU-6050 (InvenSense, [2]). Barometric pressure is measured by the LPS22HBTR (STMicroelectronics, [3]). The data sampling frequency to the micro-SD-card [4] is 5 Hz for all data except the O_2_ and pH readings that are sampled with 0.2 Hz. O_2_ and pH in the six parallel cell cultures (cf. Fig. S11) are measured using six parallel double-channel lock-in-amplifiers [5] that need for proper function a digital sinewave generator [6], a phase shifter [7], an automatic gain control [8] and a voltage converter to provide ±15V [9]. Finally, data are also available through Bluetooth [10]. In the lower right corner, the PTC-based heating element (cf. Fig. S1, [11]) is visible. Panel **B** demonstrates the indication of the device’s status on the outside.


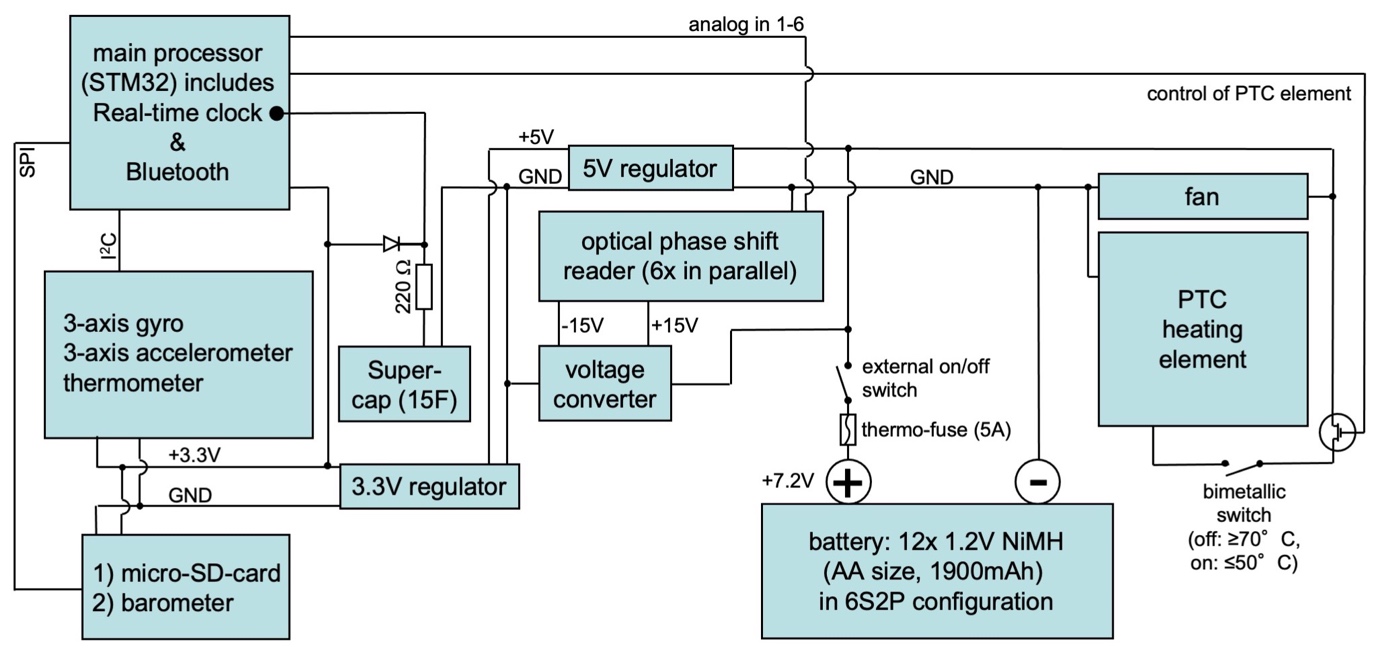


*Fig. S7.* Electronic block diagram of the cell culture device used for hypoxia experiments (shown in Fig. S6) as finally approved by the safety engineer of Novespace.


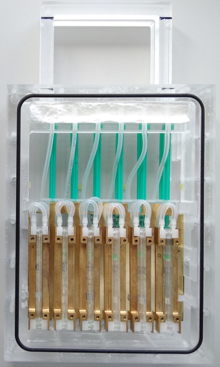


*Fig. S8.* Body of the hypoxic cell culture device, equipped with six oxygen-tight cell culture assemblies (see Fig. 1 in the main manuscript) and hold by a specifically designed brass holder. Each of the cell culture assemblies was attached to two syringes, one for inducing hypoxia with a deoxygenation solution and the other for fixing the cells with buffers for RNA or protein extraction (see below). The fluid waste is directed into a waste compartment filled with napkins as soaking material.

The heater inside the cell culture devices allows for warming the cell cultures starting from room temperature to 37°C within about 20 (device for normoxic experiments) or 5 minutes (device for hypoxia experiments). The faster warming up in the devices for hypoxic experiments is due to the brass holder because the cells were incubated and grown in the glass cell culture assemblies already mounted to the brass holder. Thus, the metal was also 37°C warm and stored heat during the time we had to keep our devices switched off (see Fig. 2 main text). Fig. S9 shows the warming-up kinetics of both device types.


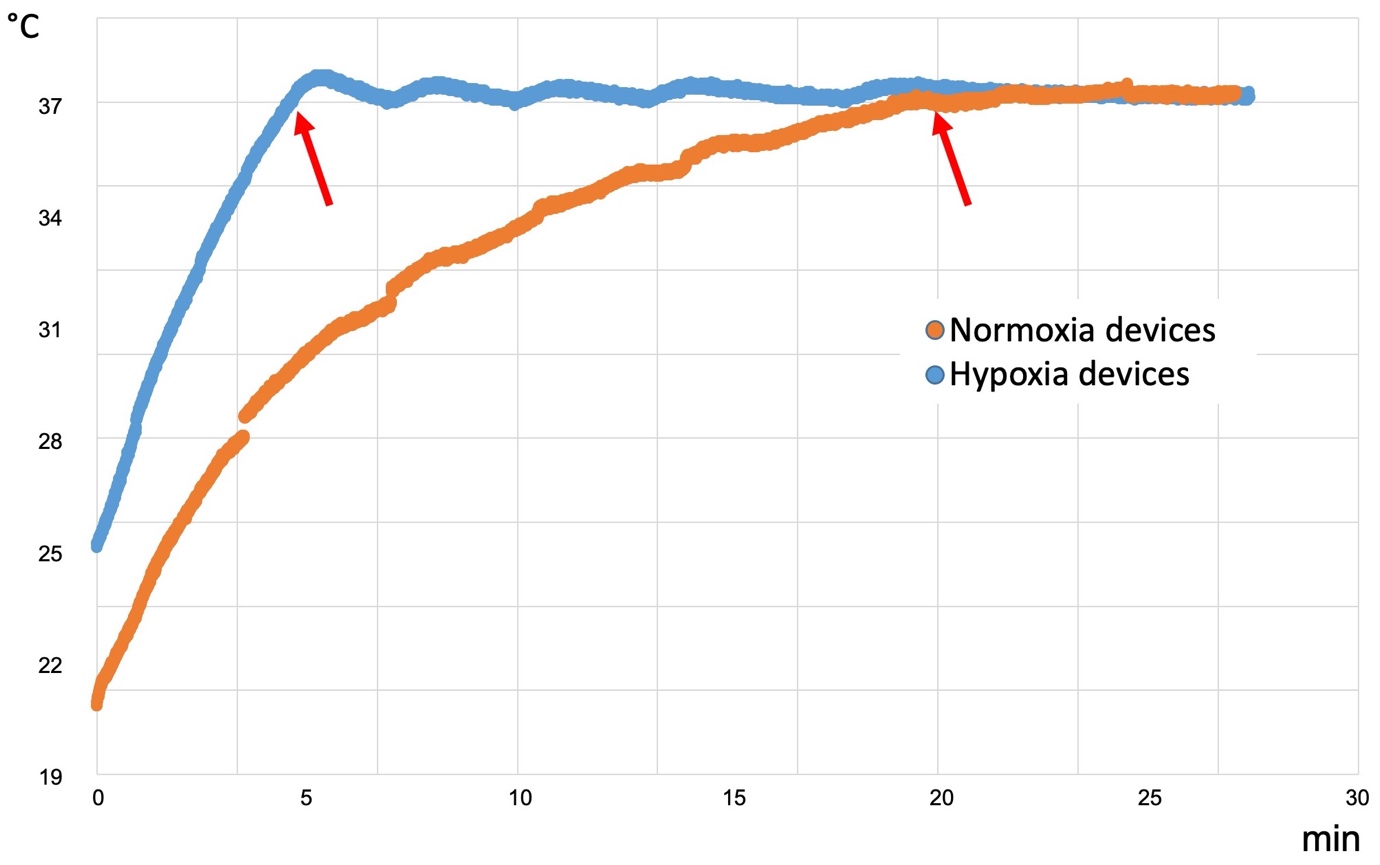


*Fig. S9.* When switching the containers on after leaving the seats, the inside temperature of devices used for hypoxic experiments (blue) was still about 25°C and not 20°C as in those used for normoxic experiments (orange) and flown and handled simultaneously. This is due to the brass mass that kept the cells warm and speeded up the warming process. The setpoint temperature was reached after 5 min in devices used for hypoxic experiments with the brass slide holder and after 20 min in devices used for normoxic experiments with plastic slide holders (red arrows).

Acceleration forces and barometric pressure data were sampled with 5Hz. The resulting traces demonstrated the precision of the MPU-6050 (Fig. S10A and B) as well as of the barometer in the hypoxia devices (LPS22HBTR) that has an accuracy of ±0.1 hPa, resulting in the detection of the deformation of the aircraft’s cabin due to the changes in gravity (Fig. S10C).


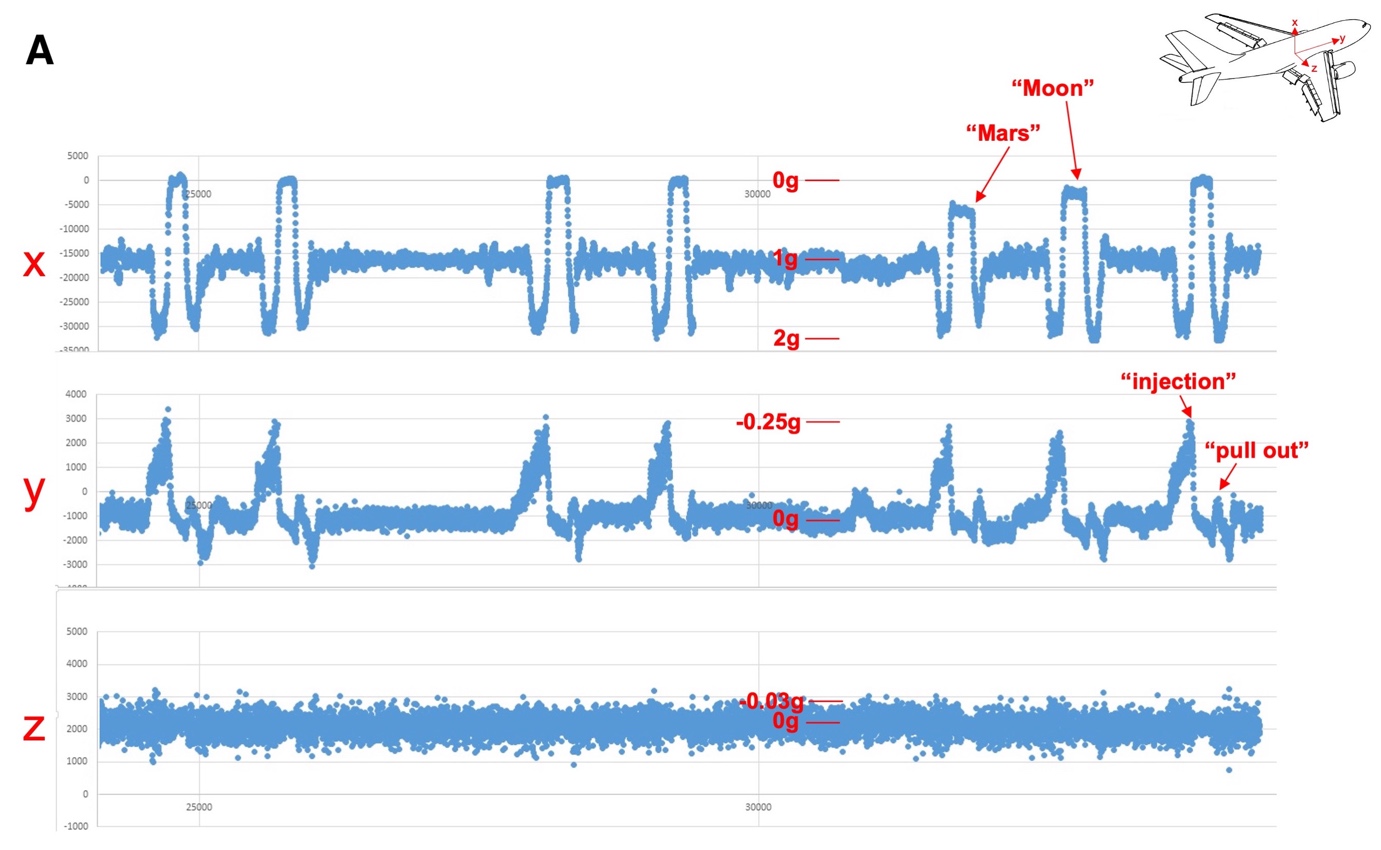


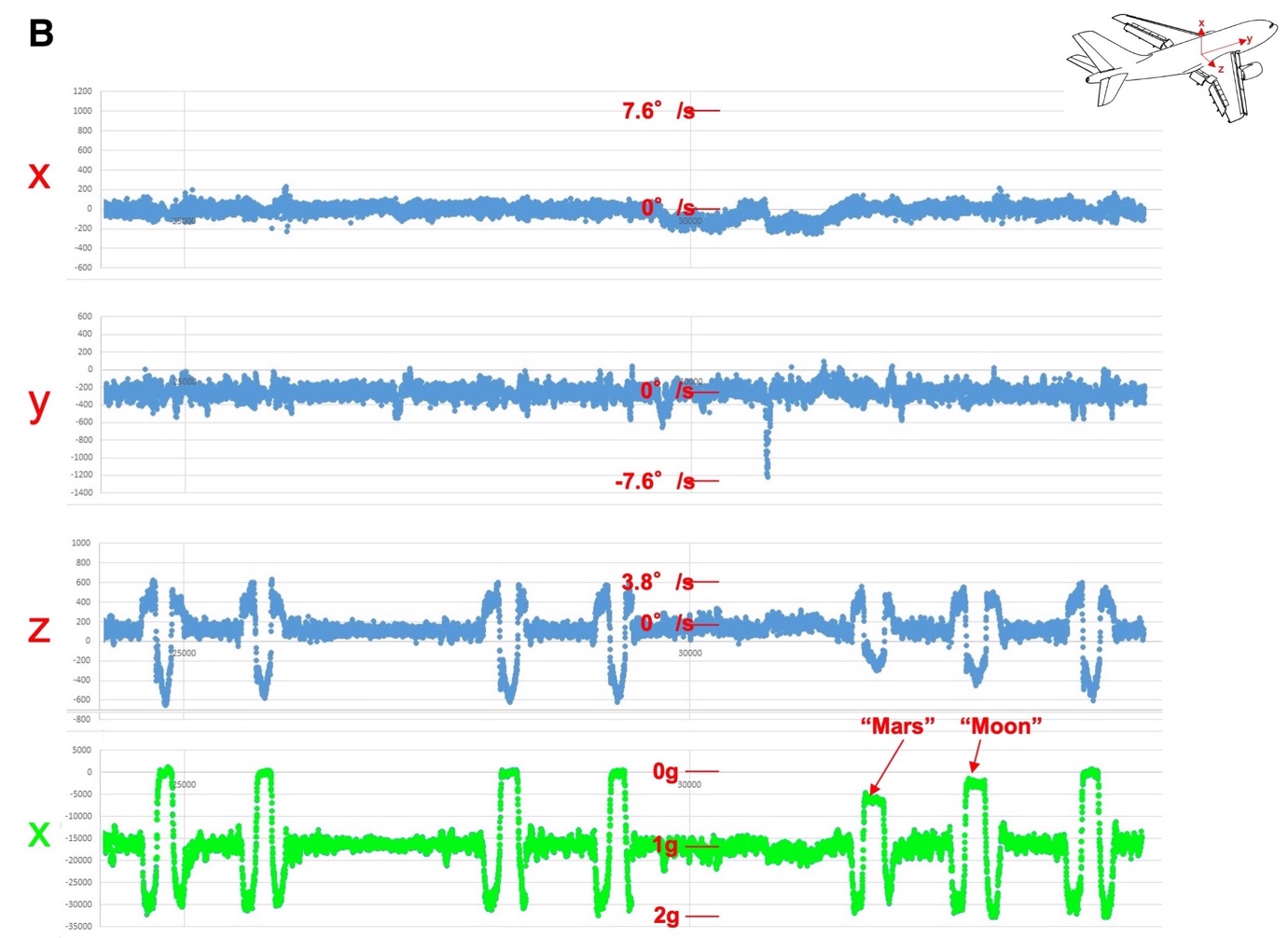


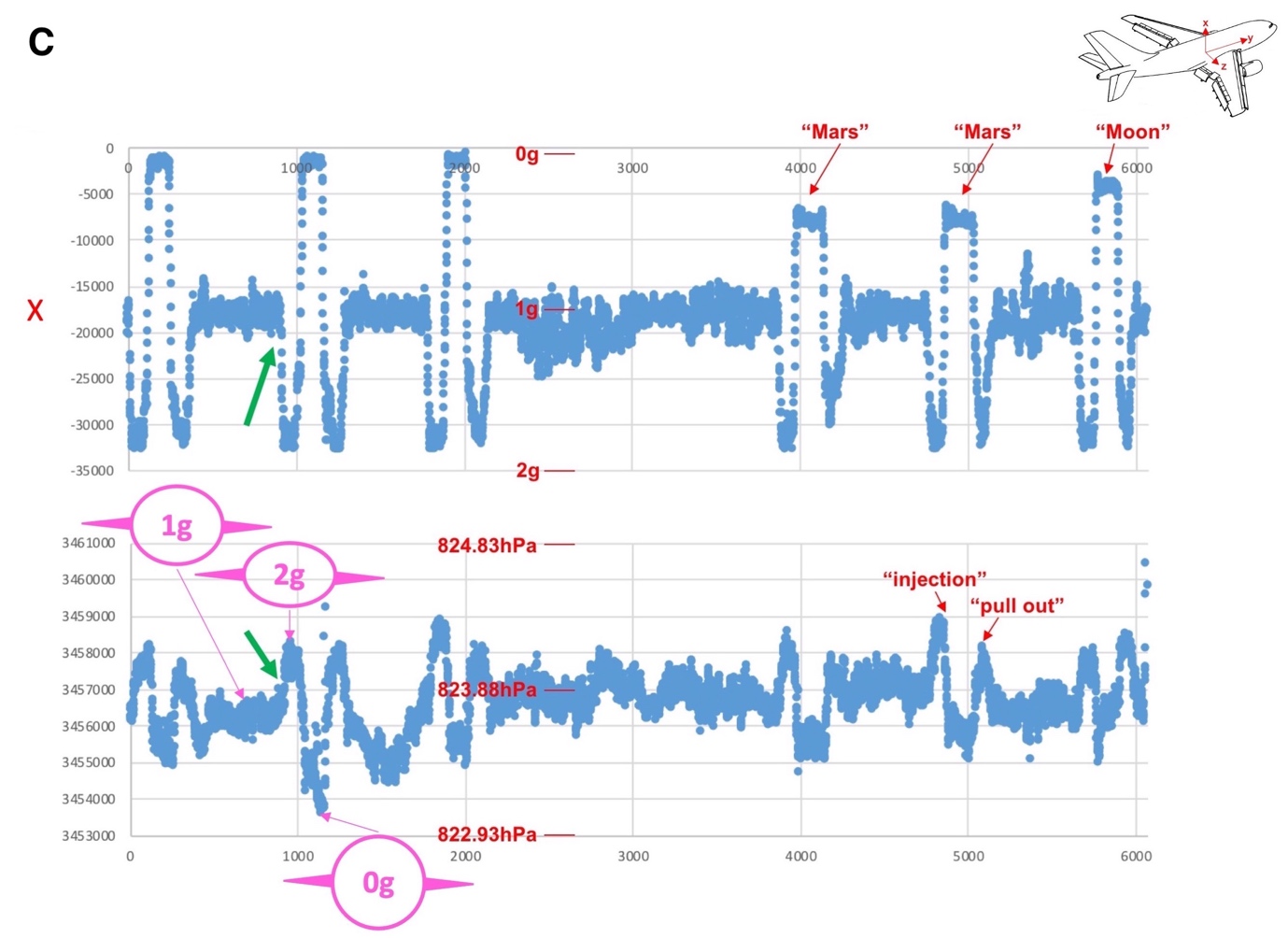


*Fig. S10.* Representative pictures of sensor traces. The insert in the upper right of each panel shows the orientation of the axes in relation to the aircraft - the x-axis is pointing to the earth’s center. Panel **A** shows the linear acceleration data. Next to the gravitational forces, acceleration forces in the nose-tail direction (Y-axis) at the beginning of each parabola were clearly detectable. Note the drop of this force at “injection” (start of the 0g- or microgravity-phase) and significant forces in nose-tail direction at “pull out” (end of the 0g- or microgravity-phase). These forces are maximally a quarter of the earth’s gravity. Finally, the vibration of the aircraft (z-trace) is about 0.03g. Panel **B** shows the angular accelerations. The elevator movements of the aircraft necessary to follow the parabolas (Z-axis, cf. also Fig. 2 main text) are clearly detectable. The green trace just correlates them to the gravitational forces in the x-direction. Panel **C** shows the barometric pressure inside the cabin in correlation to the gravitational forces. Natural gravity slightly flattens the otherwise round-shaped cabin-cross section (magenta scheme, 1g). When the aircraft is rising into the sky to start the parabola (“pull up”, green arrows), the gravity is nearly doubled which further flattens the cabin (magenta scheme, 2g) resulting in a slight increase of cabin pressure. At ”injection” the 0g-phase starts and the cabin cross-section has a perfect circular shape (magenta scheme, 0g) resulting in a barometric pressure drop below that measured at 1g.

The devices used for the hypoxic experiments were equipped with 6 parallel double channel phase shift reads or lock-in-amplifiers (LIAs) that had been designed based on a modulator/demodulator chip, namely the AD630ARZ (Analog Devices) ^1^. The details of the optics are summarized in Table S1.

The cell culture assemblies inside the devices were equipped with small, squared pieces (about 3-4mm) of pO_2_ and pH sensor foils which relied on the composition reported previously^2,3^. Briefly, the oxygen-sensitive material is based on palladium (II) meso-tetra (4-fluorophenyl) benzoporphyrin embedded into the polystyrene layer (1.5% wt. with respect to the polymer). This layer (thickness ~7 µm) is coated onto inert and transparent poly (ethylene terephthalate) support. The pH-sensitive material makes use of an aza-BODIPY dye (4-hydroxyphenyl receptor, compound 1 from reference^3^ immobilized in polyurethane hydrogel D4 along with luminescent inorganic phosphor chromium(III)-activated gadolinium aluminum borate that is added for referencing purposes (conversion of the fluorescence intensity into the phase shift measurements). Similar to the pO_2_ sensor, the composite material is coated onto poly(ethylene terephthalate) support.

The response of the materials in the devices for hypoxic measurements was compared to that obtained with a commercially available phase fluorometer Firesting from PyroScience (Aachen, Germany). Fig. S11A shows that the performance of the pO_2_ sensing material read out with the two devices is very similar. The material is particularly sensitive from 0 to about 2% O_2_ which makes it best suitable for monitoring oxygen at hypoxic conditions. Unfortunately, interrogation of the pH sensor with the home-made LIA delivered inferior results compared to the read-out with the commercially available phase fluorometer (Fig. S11B). Although the foil still shows the response to pH, the phase resolution is reduced significantly and the apparent pK_a_ value is shifted to the lower pH values (7.4 and 8.0 for homemade LIA and Firesting, respectively). Reduced sensitivity was expected because we did not measure the reference dye since we wanted to keep the set-up simple, and we were more interested in the oxygen measurements. Preliminary experiments without reading out the reference signal (not shown) revealed best phase resolution for the pH measurements by using a blue LED although the pH sensor was initially designed for excitation with red light.


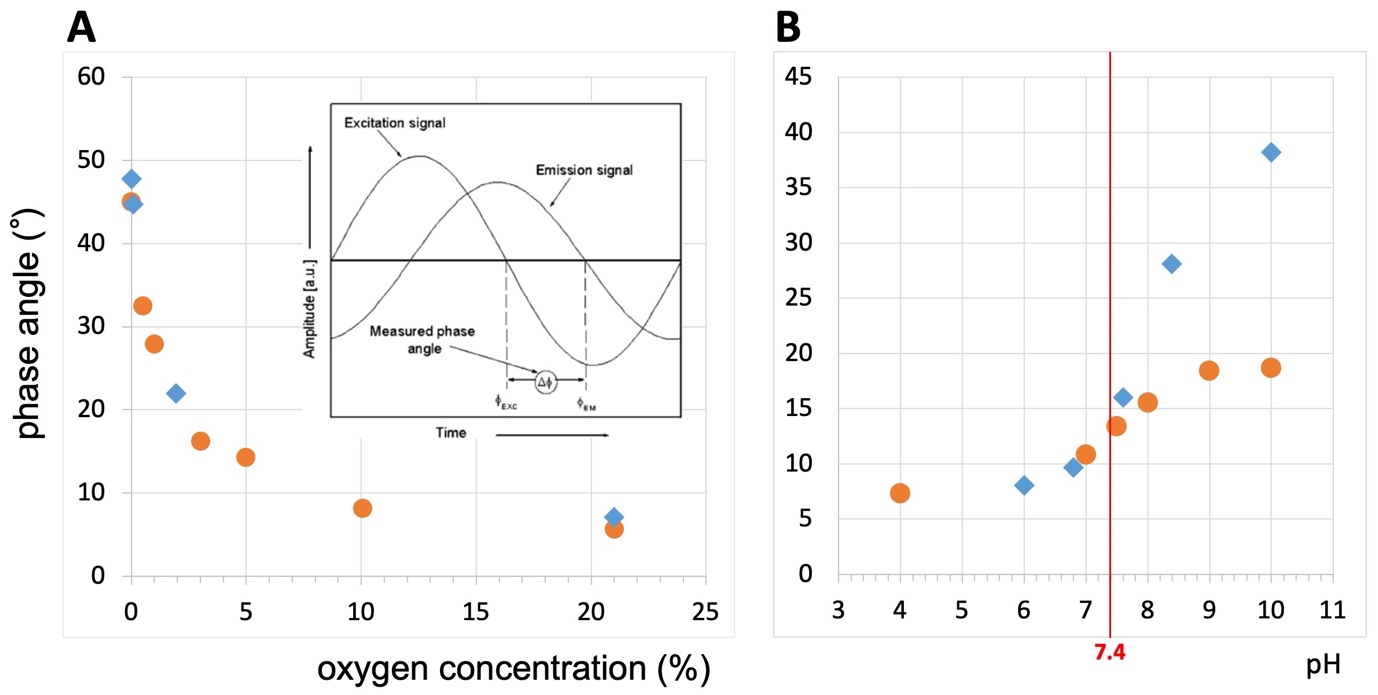


*Fig. S11* Response of the sensor materials to oxygen using calibrated gas mixtures (panel A) and pH using calibrated TRIS buffer solutions (panel B). Blue diamonds and orange dots represent data obtained with commercially available equipment and homemade LIA, respectively. Regarding pH, the sensitivity with either equipment was the highest around a pH of 7.4 (red line). The insert in panel A illustrates the measuring principle which is the phase shift determination between excitation and emission signal.

|  | **Optics for oxygen measurement** | **Optics for pH measurement** |
| --- | --- | --- |
| LED | L-7113SEC-H,  *Kingbright* | MLeROY-A1-0000-000501  (“royal blue”), *Cree Inc.* |
| LED-filter | B+W 486 UV-IR,  *Jos. Schneider Optische Werke GmbH* | B-390 UV-VIS Bandpass Filter,  *Edmund Optics Inc.* |
| Photodiode | BPW 34 FA  *Osram* | OP954  *TT Electronics* |
| Photodiode-filter | included in photodiode | RG665,  *Newport Corp.*  Primary red,  *Lee Filters* |
| Excitation frequency | 500 Hz | 2 kHz |

*Table S1.* Specifications of the optics and the parts (provider: *italic*) used for them.

To control the direction of the gravitational forces applied to the cells, and for safety reasons, the cell culture devices must be mounted to the aircraft’s floor. To this end, J.V. designed an aluminum baseplate (machined by the Physics Workshop, University of Zürich) that allows for mounting the cell culture devices either upright or lying on the aircrafts floor. This way, the gravitational forces always acted from top to bottom direction on the surface hosting the cells. Note that in the normoxia devices the surface hosting the cells is parallel to the bottom of the cell culture device whereas in the hypoxia devices it is perpendicular to it. The baseplate and its configurations are described in detail in Fig. S12.


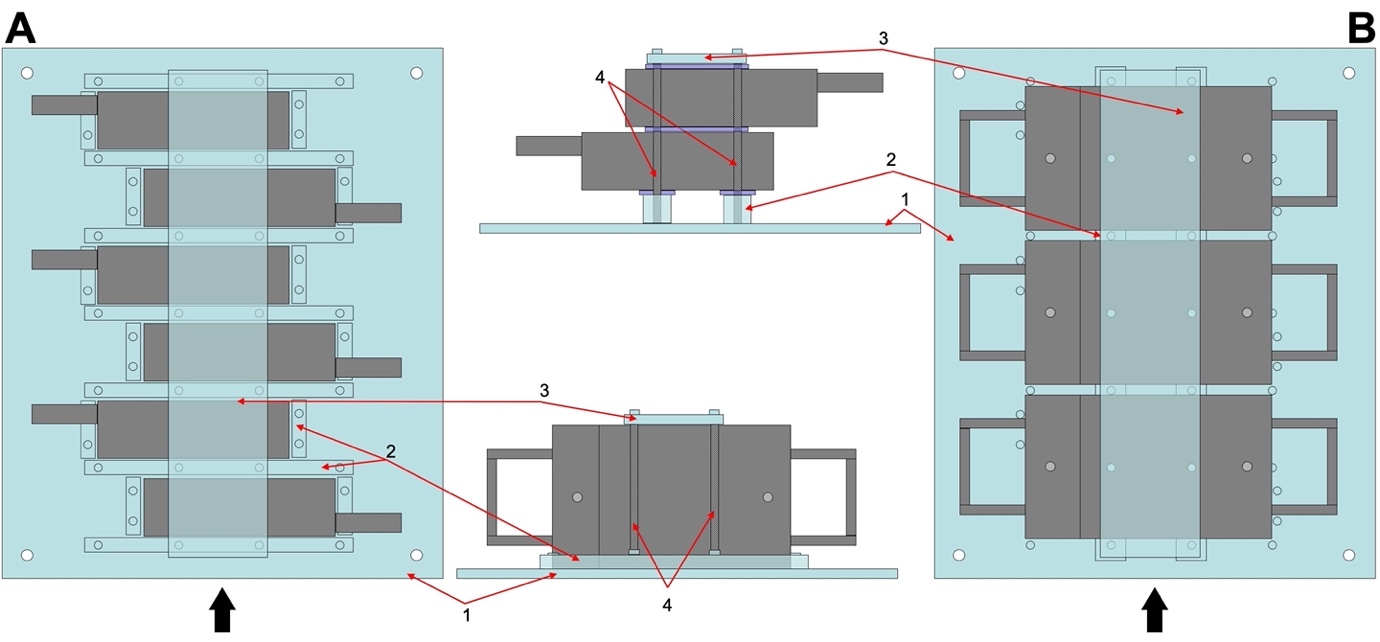


*Fig. S12.* Scheme of the upright (**A**) or lying (**B**) configuration of the cell culture devices (gray) on the 10mm thick aluminum baseplate (1). Left or right: top view, middle: side view from the direction of the black arrow. All blue circles on the top-view images indicate holes for M8 screws and white circles indicate holes for mounting the baseplate to the aircraft’s floor. In flight, the containers were individually sealed into welded plastic bags (not shown here) and the upper aluminum plate (3) was padded with pipe insulation foam. The screw strength is 6.8.

Upright mounting configuration (**A**): Each container is held by 15x15mm moldings (2) mounted to the baseplate (M8 screws, screw thread cut directly into the baseplate). From the top, the cell culture devices are held tightly in place by a 10mm aluminum plate (3) that is mounted to the base plate using 14 M8 threaded rods (4) and suitable M8 screw nuts. Lying mounting configuration (**B**): Two containers each are stacked in the way shown and three of such stacks fit on the baseplate. They are held in place by pressing them with the cover-plate (3) to 30x30mm moldings (2) that are mounted on the baseplate using 8 M8 threaded rods (4) and suitable M8 screw nuts. Anti-sliding-mats (middle, upper scheme, blue) between cell culture devices and aluminum parts exclude movements during flight.

***Deoxygenation solution***

The de-oxygenation solution consisted of 20g NaSO_3_, 1.4mg CoCl_2_, and 300µL 10M NaOH per 100mL ddH_2_O^4^. The CoCl_2_ must be dissolved first in H_2_O before adding NaSO_3_ and lastly the NaOH solution.

***Fixing solutions***

*RNA analysis:* We used the LB+TG buffer of the Promega, ReliaPrep RNA Cell Miniprep System Z6012 as described for adherent cells in section 8.A of the supplier’s kit manual. 1.5 ml 1-Thioglycerol (TG) was added to 150 ml of BL buffer. LB+TG buffer is storable at 2-10°C for up to 1 month. Finally, the BL + TG buffer contained 4M Guanidine thiocyanate, 0.01M Tris (pH7.5), and 2% 1-Thioglycerol.

*Protein analysis:* RIPA (Radio-Immunoprecipitation Assay) lysis buffer was used to lyse the cells in flight. This solution contained 50 mM Tris/HCl pH8, 150 mM NaCl, 1% NP40 (IGEPAL= NP40 substitute and BCA compatible), 0.5% Na deoxycholate, 1 mM EDTA, 0.1% SDS, 10% Glycerol (skip if you want to run BCA protein determination assay). Finally, a protease inhibitor cocktail (Cat. 539134, Calbiochem) had been added per 25 to 50 ml buffer. Shock freezing of protein solutions in liquid nitrogen and long-term storage can be performed after the addition of 10 – 20% glycerol.

*Immunocytochemistry:* For fixing the cells for HIF1α immunocytochemistry and phalloidin staining (see below), 4% formalin in phosphate-buffered saline at a pH of 7.4 was used. Cells were later stained and observed in situ in the IBIDI slides or glass cell culture assemblies.

***Immunocytochemistry against HIF1α and phalloidin staining***

After arrival in our lab in the afternoon of the parabolic flight day, the 4% formalin solution was immediately exchanged with a 2% formalin solution. On the next day, the formalin was removed, and the cells were washed three times with PBS, permeabilized for 4 min with 0.1% Triton-X-100 and washed again three times with PBS. Then, 5% normal goat serum with 0.05% Tween20 in PBS was added as a blocking solution and the slides were incubated for 60 min. After washing three times with PBS, the first antibody (HIF1α NOVUS NB100-479) was added in a dilution of 1:100 for 60 min at room temperature followed by washing three times with PBS. The secondary antibody (anti-rabbit Alexa 546, ThermoFisher) was added in a dilution of 1:800 for 60 min at room temperature. After washing three times with PBS, phalloidin 488 (Abcam) was added in a dilution of 1:400 in PBS and incubated for another 60 min. After again washing three times with PBS, DAPI counterstaining was performed for 2 min at a dilution of 1:1000 in PBS followed by washing three times with PBS. Slides were mounted with fluorescence mounting media (DAKO, S3023).

***Western blotting***

After collecting the RIPA protein sample, proteins were precipitated by adding 5 volumes of -20°C cold acetone to the sample, vortexing, incubating for 30 min at -20°C and centrifuging for 5 min at 6000 – 10000g. Then the supernatant was removed, the pellet was air-dried and then resuspended in RIPA buffer. After adding 4x Lämmli buffer, the sample was boiled for 5 min at 95°C and loaded on SDS-PAGE gel 10%. After transfer to nitrocellulose blotting membrane (GE Healthcare, #10600002), the membrane was blocked in 5% BSA in TBST for 1h at RT. Primary antibodies (anti- β-Actin mouse IgG A5441 Sigma-Aldrich; 1:8000 and HIF-1α NB100-479 NovusBio; 1:100) were incubated overnight at 4°C. After rinsing the membrane 3-5 times each for 5 min with TBST, it was incubated with an HRP-conjugated secondary antibody solution: donkey-anti-rabbit 1:5000 (Amersham, NA934V) for HIF-1α or goat-anti-mouse 1:5000 (Santa Cruz, sc-2031) for β-Actin, for 60 min at room temperature. After rinsing the membrane 3-5 times 5 min with TBST, the chemiluminescent substrate (SuperSignal / Thermo Scientific) was applied to the blot and the signal was captured using a CCD camera-based imager (ChemiDoc TM Imaging System, BioRad). We could not provide the full-length membranes for some plots because the membranes were cut before hybridization with the primary antibody. We avoid stripping/re-staining the membranes as much as we can to avoid the misleading band ghosts from the first hybridization (in case of weak stripping) or weak bands from the second hybridization (in case of strong stripping). Therefore, whenever possible, we cut the membrane at the corresponding band size, according to our initial testing and the antibody datasheet. Moreover, cutting membranes was necessary as we can’t run as many gels due to limited samples volume.

***RNA extraction and real-time PCR***

RNA extraction was performed using ReliaPrep RNA Cell Miniprep System (Promega, Z6011), according to the manufacturer. First-strand cDNA was synthesized following the manufacturer’s protocol using the RevertAid First Strand cDNA Synthesis Kit (Thermo Scientific, K1622). A final cDNA concentration of 5ng/µl was used for semi-quantitative real-time PCR analysis performed in Thermocycler ABI7500 Fast (Applied Biosystems), using PowerUp SYBR Green Master Mix (Applied Biosystems, A25778). Primers were designed with Primer3 and are listed in (Table S2). Primers were validated first by qRT-PCR via i) melting curve analyses (mode integrated into the 7500 Fast Real-Time PCR System to confirm having one single peak corresponds to desired product) as well as ii) on acrylamide gels to confirm size and purity of PCR products (Fig. S14) iii) Sanger sequencing of PCR products. DDCt method was used to calculate mRNA expression levels^6,7^.

| Target gene | Primers Sequences |
| --- | --- |
| ACTB | *F: 5’-CTGGAACGGTGAAGGTGACA-3’*  *R: 5’-AAGGGACTTCCTGTAACAACGA-3’* |
| CITED2 | *F: 5'-ACCATCACCCTGCCCACC-3'*  *R: 5'-CGTAGTGTATGTGCTCGCCCA-3'* |
| EGLN1 | *F: 5'-CAAATGGAGATGGAAGATGTGTG-3'*  *R: 5'-AATGTCAGCAAACTGGGCTTT-3'* |
| EPO | *F: 5'-ATGTGGATAAAGCCGTCAGT-3'*  *R: 5'-AGTGATTGTTCGGAGTGGAG-3'* |
| GLUT1 | *F: 5'-TCTGGCATCAACGCTGTCTTC-3'*  *R: 5'-CGATACCGGAGCCAATGGT-3'* |
| LDHA | *F: 5'-ATGGCAACTCTAAAGGATCAGC-3'*  *R: 5'-CCAACCCCAACAACTGTAATCT-3'* |
| OCT4 | *F: 5'-CTTGAATCCCGAATGGAAAGGG-3'*  *R: 5'-CCTTCCCAAATAGAACCCCCA-3'* |
| VEGFA | *F: 5'-AGGGCAGAATCATCACGAAGT-3'*  *R: 5'-AGGGTCTCGATTGGATGGCA-3'* |

*Table S2.* Forward (F) and reverse (R) primers used for PCR analysis of the listed target genes.

ACTB = beta actin, CITED2 = CBP/p300 interacting transactivator with ED-rich tail 2, EGLN1 = Egl-9 Family Hypoxia Inducible Factor 1 (=PHD2), EPO = erythropoietin, GLUT1 = glucose transporter type 1, LDHA = Lactate Dehydrogenase A, OCT4 = POU transcription factor, VEGF = vascular endothelial growth factor


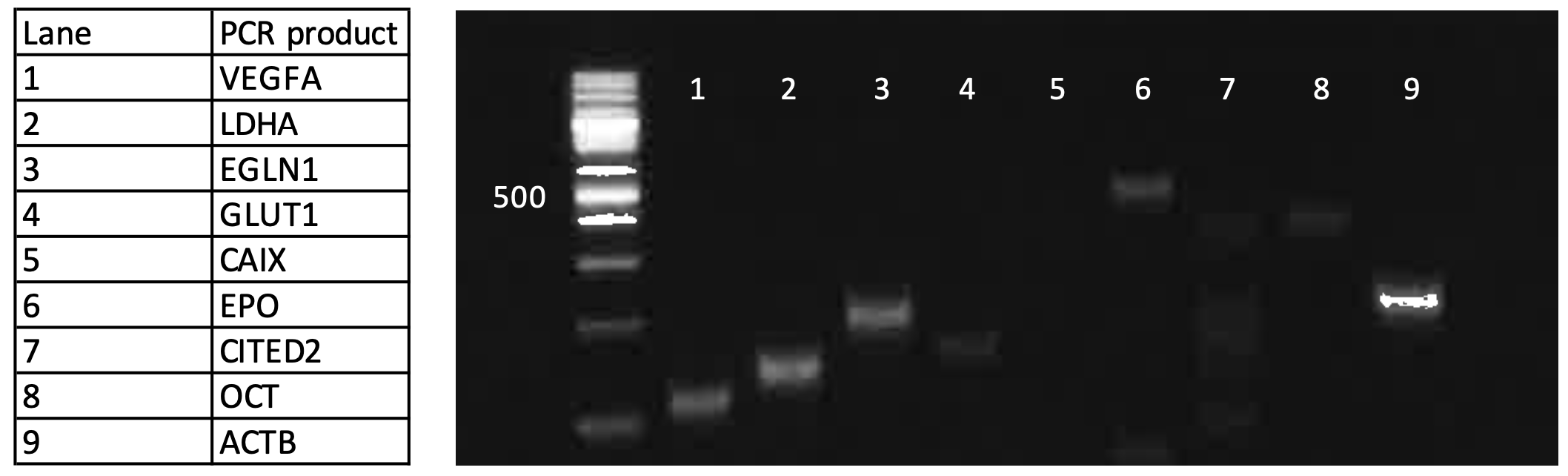


*Fig. S14.* Validation of the primers on acrylamide gels. Size and purity of PCR products is as expected.

**References:**

1. Sengupta S, Farnham J, Whitten J. A simple low-cost lock-in amplifier for the laboratory. *J Chem Education*. 2005;82:1399-1401.

2. Borisov SM, Nuss G, Klimant I. Red light-excitable oxygen sensing materials based on platinum(II) and palladium(II) benzoporphyrins. *Anal Chem*. 2008;80:9435-9442. doi: 10.1021/ac801521v

3. Jokic T, Borisov SM, Saf R, Nielsen DA, Kühl M, Klimant I. Highly photostable near-infrared fluorescent pH indicators and sensors based on BF2-chelated tetraarylazadipyrromethene dyes. *Anal Chem*. 2012;84:6723-6730. doi: 10.1021/ac3011796

4. Vanderhorst R, Lewis SD. Potential of sodium sulfite catalyzed with cobald chloride in harvesting fish. *The Progressive Fish-culturist*. 1969:149-154.

5. Gassmann M, Grenacher B, Rohde B, Vogel J. Quantifying Western blots: pitfalls of densitometry. *Electrophoresis*. 2009;30:1845-1855. doi: 10.1002/elps.200800720

6. Livak KJ, Schmittgen TD. Analysis of relative gene expression data using real-time quantitative PCR and the 2(-Delta Delta C(T)) Method. *Methods*. 2001;25:402-408. doi: 10.1006/meth.2001.1262

7. Pfaffl MW. A new mathematical model for relative quantification in real-time RT-PCR. *Nucleic Acids Res*. 2001;29:e45. doi: 10.1093/nar/29.9.e45
